# PIMMS43 is required for malaria parasite immune evasion and sporogonic development in the mosquito vector

**DOI:** 10.1073/pnas.1919709117

**Published:** 2020-03-12

**Authors:** Chiamaka V. Ukegbu, Maria Giorgalli, Sofia Tapanelli, Luisa D. P. Rona, Amie Jaye, Claudia Wyer, Fiona Angrisano, Andrew M. Blagborough, George K. Christophides, Dina Vlachou

**Affiliations:** ^a^Department of Life Sciences, Imperial College London, SW7 2AZ London, United Kingdom

**Keywords:** malaria transmission, mosquito innate immunity, complement-like response, transmission blocking vaccines, mosquito population replacement

## Abstract

After being ingested by a female *Anopheles* mosquito during a bloodmeal on an infected host, and before they can reach the mosquito salivary glands to be transmitted to a new host, *Plasmodium* parasites must establish an infection of the mosquito midgut in the form of oocysts. To achieve this, they must first survive a series of robust innate immune responses that take place prior to, during, and immediately after ookinete traversal of the midgut epithelium. Understanding how parasites may evade these responses could highlight new ways to block malaria transmission. We show that an ookinete and sporozoite surface protein designated as PIMMS43 (*Plasmodium* Infection of the Mosquito Midgut Screen 43) is required for parasite evasion of the *Anopheles coluzzii* complement-like response. Disruption of PIMMS43 in the rodent malaria parasite *Plasmodium berghei* triggers robust complement activation and ookinete elimination upon mosquito midgut traversal. Silencing components of the complement-like system through RNAi largely restores ookinete-to-oocyst transition but oocysts remain small in size and produce a very small number of sporozoites that additionally are not infectious, indicating that PIMMS43 is also essential for sporogonic development in the oocyst. Antibodies that bind PIMMS43 interfere with parasite immune evasion when ingested with the infectious blood meal and significantly reduce the prevalence and intensity of infection. PIMMS43 genetic structure across African *Plasmodium falciparum* populations indicates allelic adaptation to sympatric vector populations. These data add to our understanding of mosquito–parasite interactions and identify PIMMS43 as a target of malaria transmission blocking.

## Significance

Malaria is transmitted among humans through mosquito bites. Here, we characterize a protein found on the surface of mosquito stages of malaria parasites and reveal that it serves to evade the mosquito immune system and ensure disease transmission. Neutralization of PIMMS43 (*Plasmodium* Infection of the Mosquito Midgut Screen 43), either by eliminating it from the parasite genome or by preincubating parasites with antibodies that bind to the PIMMS43 protein, inhibits mosquito infection with malaria parasites. Differences in PIMMS43 detected between African malaria parasite populations suggest that these populations have adapted for transmission by different mosquito vectors that are also differentially distributed across the continent. We conclude that targeting PIMMS43 can block malaria parasites inside mosquitoes before they can infect humans.

Enhanced vector control significantly reduced malaria cases in recent years and, together with effective medicines and better health care, decreased the number of malaria-associated deaths. However, the effectiveness of these measures is currently compromised due to widespread mosquito resistance to insecticides used in bed-net impregnation and indoor residual spraying, while mosquito biting and resting behaviors have also changed in response to these measures. As a result, no significant progress in reducing the global malaria burden is recorded in the past years. Therefore, additional tools for malaria control are needed, the development of which could be guided by a better understanding of disease transmission through the vector.

Mosquito acquisition of *Plasmodium* parasites commences when a female *Anopheles* mosquito ingests gametocyte-containing blood from an infected person. In the mosquito midgut lumen, gametocytes mature and produce gametes. Fertilization of gametes leads to zygotes that soon develop to ookinetes and traverse the midgut epithelium. At the midgut basal subepithelial space, ookinetes differentiate into replicative oocysts wherein hundreds of sporozoites develop within a period of 1 to 2 wk. Upon release into the hemocoel, sporozoites—transported by the hemolymph—traverse the salivary glands and infect a new host upon a next mosquito bite.

Inside the mosquito, parasites are attacked by an array of immune responses (1, 2). Most parasite losses occur during the ookinete-to-oocyst transition (3, 4). Ookinete traversal of the mosquito midgut leads to activation of JNK (c-Jun N-terminal kinase) signaling, inducing apoptosis of the invaded cells. This response involves various effectors, including heme peroxidase 2 and NADPH oxidase 5 that potentiate nitration of ookinetes that are henceforth marked for elimination by reactions of the mosquito complement-like system (5, 6). These reactions are triggered upon ookinete exit at the midgut subepithelial space encountering the hemolymph that carries the complement-like system.

The hallmark of the mosquito complement-like system is the C3-like factor, TEP1 (7, 8). A proteolytically processed form of TEP1, TEP1_cut_, circulates in the hemolymph as a complex with LRIM1 and APL1C (9, 10). Upon parasite recognition, TEP1_cut_ is released from the complex and attacks the ookinete, triggering in situ assembly of a TEP1 convertase that locally processes TEP1 molecules that bind to the ookinete causing lysis and, in some cases, melanization (11). These reactions are regulated by CLIP-domain serine proteases and their inactive homologs (11, 12). Ookinete clearance is assisted by actin-mediated cellular responses of invaded epithelial cells (13).

The characterization of *Plasmodium falciparum* Pfs47 as a player in parasite evasion of the mosquito complement-like response has opened new avenues to dissect the mechanisms parasites employ to endure or indeed evade the mosquito immune response. glycosyl-phosphatidylinositol (GPI)-anchored Pfs47 was hypothesized to interfere with activation of JNK signaling and, by doing so, aids ookinetes to escape nitration and subsequent complement-mediated attack (14, 15). This parasite immune evasion function is shared by the Pfs47 ortholog in the rodent malaria parasite *Plasmodium berghei* (16), which was earlier thought to be solely involved in fertilization (17).

Our transcriptomic profiling of field *P. falciparum* isolates from Burkina Faso in the midgut of sympatric *Anopheles coluzzii* (previously *Anopheles gambiae* M form) and *Anopheles arabiensis* mosquitoes and a laboratory *P. berghei* strain in the midgut of *A. coluzzii* (18) identified hundreds of genes exhibiting conserved and differential expression during gametocyte-to-oocyst development. Several of them encoding putatively secreted or membrane-associated proteins were made part of a screen to identify genes that function during parasite infection of the mosquito midgut. These genes were given a candidate gene number preceded by the acronym *PIMMS*, for “*Plasmodium* Infection of the Mosquito Midgut Screen.” We previously characterized *PIMMS2* that encodes a subtilisin-like protein involved in midgut traversal (19). Here, we report the characterization of *P. falciparum* and *P. berghei PIMMS43* that encodes a membrane-bound protein found on the surface of ookinetes and sporozoites. The gene was first reported in *P. berghei* to be a target of the transcription factor AP2-O, has a role in mosquito midgut invasion and oocyst development, and was named *POS8* (20). A later study by another group reported the gene as being important for ookinete maturation, designating it as *PSOP25* (21). Here we demonstrate that *PIMMS43* has no detectable function in ookinete maturation or mosquito midgut invasion but plays a key role in ookinete evasion of the mosquito complement-like response. We show that disruption of *PIMMS43* leads to robust complement activation and ookinete elimination upon completion of midgut traversal and before their transformation to oocysts. When the complement system is inactivated, oocyst transformation is restored but sporogony cannot be completed, as the gene is also essential for sporozoite development. Parallel analysis of thousands of African *P. falciparum* parasites reveals clear genetic differentiation between populations sampled from West or Central and East African countries, inferring parasite adaptation to sympatric vector populations. We further demonstrate that *A. coluzzii* ingestion of antibodies against *P. falciparum* PIMMS43 leads to strong inhibition of oocyst development. The discovery and characterization of PIMMS43 adds to our understanding of parasite immune evasion and malaria transmission through the vector.

## Results and Discussion

### Identification of *PIMMS43*.

*P. falciparum* (*PF3D7_0620000*) and *P. berghei* (*PBANKA_1119200*) *PIMMS43* encode deduced proteins of 505 and 350 amino acids, respectively. N-terminal signal peptides (amino acids 1 to 25 for PfPIMMS43 and 1 to 22 for PbPIMMS43) and C-terminal transmembrane domains (amino acids 482 to 504 for PfPIMMS43 and 327 to 350 for PbPIMMS43) are predicted for both proteins. The transmembrane domains are predicted by PredGPI to also contain signals for attachment of a GPI lipid anchor with 99% probability.

PIMMS43 is conserved among species of the *Plasmodium* genus. All orthologs are predicted to contain the N-terminal signal peptide and C-terminal transmembrane domain, as well as a conserved pair of cysteine residues adjacent to the C terminus (*SI Appendix*, Fig. S1). PbPIMMS43 exhibits a 68% sequence identity with orthologs in other rodent parasites (i.e., *Plasmodium yoelii* and *Plasmodium chabaudi*) and 27% and 24% with *P. falciparum* and *Plasmodium vivax* PIMMS43, respectively. PfPIMMS43 and PvPIMMS43 contain a 60 to 180 nonconserved amino acid insertion with no obvious sequence similarity between them, which are therefore likely to have occurred independently. Another shorter, nonconserved insertion toward the C terminus of *P. vivax* and *Plasmodium knowlesi* PIMMS43 includes tandem repeats of Glycine-Serine-Glutamine-Alanine-Serine (GSQAS).

### *PIMMS43* Transcripts Peak in Ookinetes and Sporozoites.

**Fig. 1.**
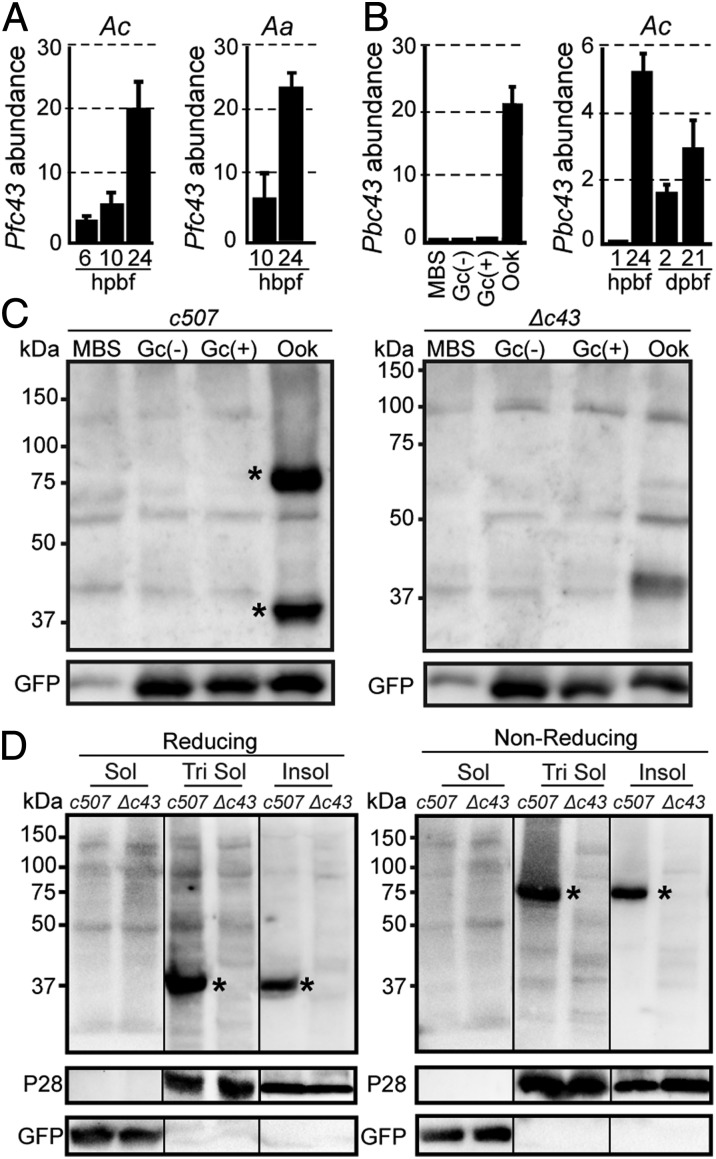
PIMMS43 transcription profiles and protein expression. (*A*) DNA microarray transcriptional profiling of *Pfc43* in *A. coluzzii* (*Ac*) and *A. arabiensis* (*Aa*) midguts. Bars show transcript abundance at indicated time points relative to 1 hpbf and are average of three biological replicates. Error bars show SEM. (*B*) Relative abundance of *Pbc43* transcripts in blood stages, in vitro ookinetes, and *A. coluzzii* mosquito stages, as determined by qRT-PCR in the *c507* line and normalized against the constitutive expressed *GFP*. Each bar is the average of three biological replicates. Error bars show SEM. (*C*) Western blot analysis under reducing conditions (3% [vol/vol] 2-mercapthoethanol) using the α-Pbc43^opt^ antibody on whole-cell lysates of *c507* parasites. Pbc43 protein bands are indicated with asterisks. *Δc43* parasites were used as a negative control. GFP was used as a loading control. (*D*) Western blot analysis under reducing (*Left*; 5% [vol/vol] 2-mercapthoethanol) and nonreducing (*Right*) conditions using the α-Pbc43^opt^ antibody on fractionated in vitro ookinetes. Pbc43 protein bands are indicated with asterisks. *Δc43* ookinetes were used as a negative control. P28 and GFP were used as stage-specific and loading controls, respectively. Soluble (Sol), Triton soluble (Tri Sol), and insoluble (Insol) fractions are shown. Abbreviations: Gc(−), nonactivated gametocytes; Gc(+), activated gametocytes; Ook, ookinetes.

DNA microarray profiling of *A. coluzzii* and *A. arabiensis* midguts infected with *P. falciparum* field isolates in Burkina Faso revealed that *PfPIMMS43* (referred to in figures as *Pfc43*) shows progressively increased transcription that peaks 24 h postmosquito blood feeding (hpbf) (Fig. 1*A*). These data were corroborated by laboratory *P. falciparum* NF54 infections of *A. coluzzii* using RT-PCR (*SI Appendix*, Fig. S2*A*). Low levels of *PfPIMMS43* transcripts were also detected in in vitro-cultured gametocytes but not in in vitro-cultured asexual blood stage (ABS) parasites, indicating that *PfPIMMS43* transcription begins in gametocytes and peaks in zygotes and ookinetes. Transcripts were not detected in oocysts 10 d postmosquito blood feeding (dpbf) but reappeared in mosquito salivary glands, indicative of *PfPIMMS43* reexpression in sporozoites. While we do not detect *PfPIMMS43* transcripts at 10 dpbf, two published RNA sequencing experiments have shown that *PfPIMMS43* transcripts are detected at 7 (22) and 8 dpbf (23) in the midgut oocysts, suggesting that *PfPIMMS43* transcripts may be too low to be detected at 10 dpbf in our assays.

We examined whether the *P. berghei PIMMS43* ortholog (referred to in figures as *Pbc43*) shows expression profile similar to *PfPIMMS43*, using quantitative real-time RT-PCR (qRT-PCR) (Fig. 1*B*) and RT-PCR (*SI Appendix*, Fig. S2*B*). In these assays, we used the *P. berghei* line *ANKA507m6cl1* that constitutively expresses GFP (24), hereafter referred to as *c507*, as well as the nongametocyte producing ANKA 2.33 (NGP) as a control in the RT-PCR assay. The results revealed low levels of *PbPIMMS43* transcripts in mixed blood stages (MBS) and purified *c507* gametocytes, which together with absence of transcripts from NGP MBS indicated that *PbPIMMS43* transcription begins in gametocytes, similar to *PfPIMMS43*. Also similar to *PfPIMMS43*, *PbPIMMS43* transcript levels were very high 24 hpbf, as well as in purified in vitro-produced ookinetes, indicating high *PbPIMMS43* transcription in ookinetes. Lower transcript levels were detected 2 dpbf, presumably due to ookinetes retained in the blood bolus and the midgut epithelium and low-level expression in young oocysts. No *PbPIMMS43* transcripts were detected in mature oocysts 10 dpbf, but strong *PbPIMMS43* reexpression was observed in salivary gland sporozoites. Taken together, these data indicate that *P. falciparum* and *P. berghei PIMMS43* exhibit similar transcription patterns starting in gametocytes, peaking in ookinetes, pausing in oocysts, and restarting in salivary gland sporozoites.

To investigate PbPIMMS43 protein expression, we raised rabbit polyclonal antibodies against a codon-optimized fragment of the protein (amino acids 22 to 327) expressed in *Escherichia coli* cells (α-Pbc43^opt^), and a native protein fragment (amino acids 22 to 331) expressed in insect *Spodoptera frugiperda* Sf9 cells (α-Pbc43^Sf9^). Both recombinant proteins lacked the predicted signal peptide and C-terminal transmembrane domain. We also generated a genetically modified *c507 P. berghei* line, designated *Δc43*, where 50% of the *PbPIMMS43* coding region was replaced with a modified *Toxoplasma gondii* pyrimethamine-resistance expression cassette (*TgDHFR*) (*SI Appendix*, Fig. S3*A*). Integration of the disruption cassette was confirmed by PCR and pulse field gel electrophoresis (*SI Appendix*, Fig. S3 *B* and *C*). RT-PCR assays confirmed that *PbPIMMS43* transcripts could no longer be detected in gametocytes, ookinetes, and sporozoites of the *Δc43* line that was henceforth used as a negative control in protein-expression experiments (*SI Appendix*, Fig. S2*B*). Western blot analysis was performed in total, triton-soluble protein extracts prepared under reducing conditions from MBS, purified gametocytes, and in vitro-cultured ookinetes of the *c507* and *Δc43 P. berghei* lines (Fig. 1*C*). Two clear bands of ∼37 and 75 kDa were detected in ookinete extracts of the *c507* line. The former band matches the predicted molecular weight of PbPIMMS43 monomer and the latter band could correspond to PbPIMMS43 dimer, either a homodimer formed upon disulfide bonding of the conserved pair of cysteine residues or a heterodimer. Indeed, under strong reducing conditions, the 75 kDa was resolved in a single 37-kDa band, whereas under nonreducing conditions only the 75 kDa band could be detected (Fig. 1*D*). This assay was combined with membrane-fractionation of total in vitro ookinete extracts, which revealed that both bands were only observed in the insoluble fraction and the fraction solubilized by triton, but not in the soluble (triton nontreated) fraction. These data indicate membrane association of PbPIMMS43, in accordance with the prediction of a transmembrane domain and a GPI anchor.

We also raised a rabbit polyclonal antibody against a codon-optimized coding fragment of *P. falciparum* PIMMS43 (amino acids 25 to 481) expressed in *E. coli* cells and lacking the predicted signal peptide and C-terminal transmembrane domain (α-Pfc43^opt^). We examined the affinity and specificity of this antibody by generating and using a *P. berghei c507* transgenic line (*Pb*^*Pfc43*^), where *PbPIMMS43* was replaced by *PfPIMMS43* (*SI Appendix*, Fig. S4*A*). PCR genotypic analysis confirmed successful modification of the endogenous *PbPIMMS43* genomic locus (*SI Appendix*, Fig. S4*B*), and RT-PCR analysis confirmed that *PfPIMMS43* is transcribed in in vitro-cultured *P. berghei* ookinetes (*SI Appendix*, Fig. S4*C*). Western blot analysis of total protein extracts prepared from purified in vitro-cultured *Pb*^*Pfc43*^ ookinetes using the α-Pfc43^opt^ antibody revealed a strong band of ∼60 kDa, corresponding to the predicted molecular weight of the deduced PfPIMMS43 protein (*SI Appendix*, Fig. S4*D*). This band was absent from *c507* and *Δc43* protein extracts, confirming the specificity of the α-Pfc43^opt^ antibody. It is noteworthy that, in contrast to what was observed with the PbPIMMS43 protein, the results did not show dimerization of the ectopically expressed PfPIMMS43 protein when the analysis was done under nonreducing conditions (*SI Appendix*, Fig. S4*D*). While this may represent a true difference between the *P. berghei* and *P. falciparum* PIMMS43 proteins, we cannot rule out the possibility that PfPIMMS43 dimerization requires, in addition to the conserved cysteine residues, a *P. falciparum*-specific cofactor that is not present in *P. berghei* and may be related to the nonconserved N-terminal amino acid insertion in PfPIMMS43.

### PIMMS43 Protein Is Localized on the Parasite Membrane.

**Fig. 2.**
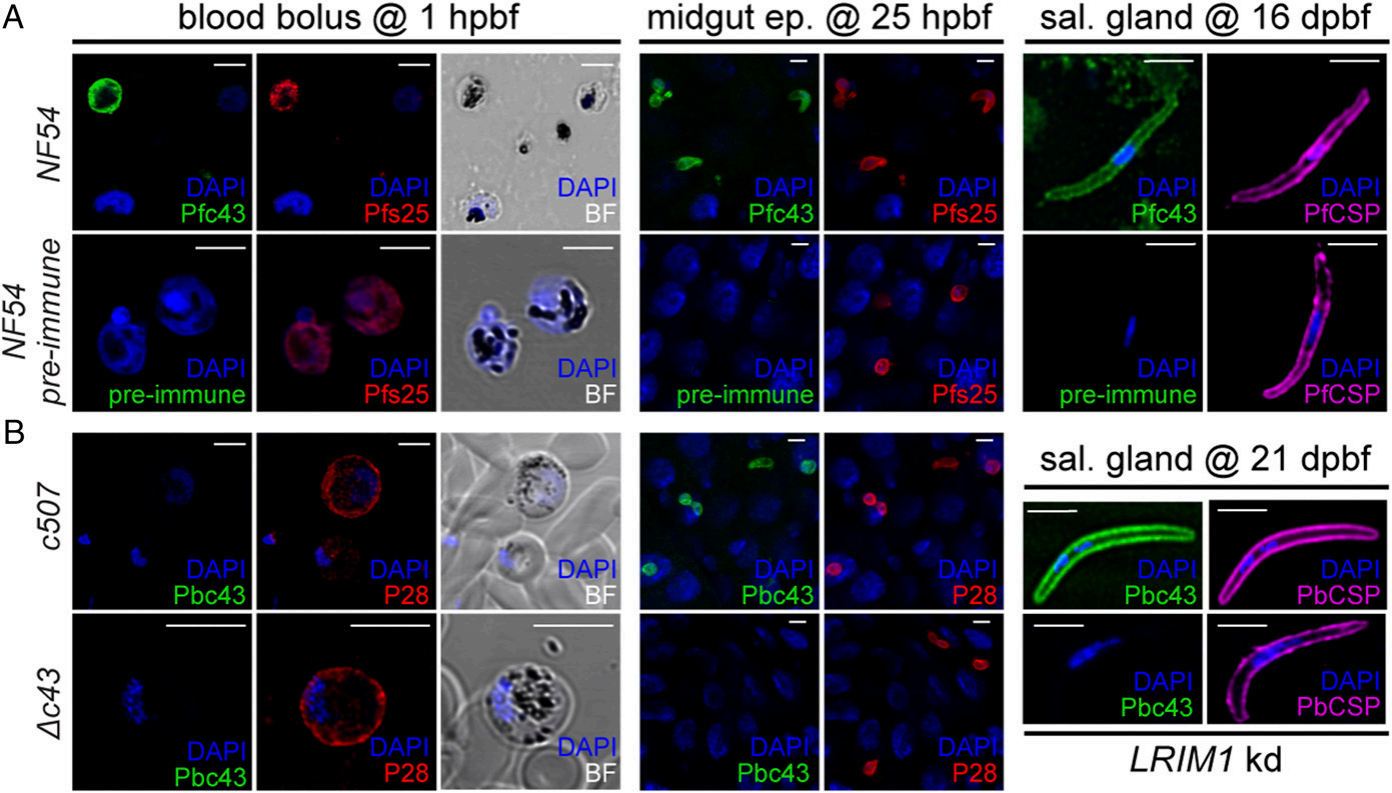
PIMMS43 protein localization. (*A*) Immunofluorescence assays of *P. falciparum* NF54 parasites found in mosquito blood bolus of at 1 hpbf (*Left*), ookinetes traversing the mosquito midgut epithelium at 25 hpbf (*Center*), and salivary gland sporozoites at 16 dpbf (*Right*), stained with α-Pfc43^opt^ (green) and the female gamete/zygote/ookinete α-Pfs25 (red) or sporozoite α-PfCSP (purple) antibodies. DNA was stained with DAPI. Staining with preimmune serum was used as a negative control. (*B*) Immunofluorescence assays of *P. berghei 507* early sexual stages (activated gametocytes and/or early zygotes) in mosquito blood bolus at 1 hpbf (*Left*), ookinetes traversing the mosquito midgut epithelium at 25 hpbf (*Center*), and salivary gland sporozoites at 21 dpbf (*Right*), stained with α-Pbc43^opt^ (green), female gamete/zygote/ookinete surface α-P28 (red) or sporozoite surface α-PbCSP (purple) antibodies. DNA was stained with DAPI. Staining of the *Δc43* parasite with α-Pbc43^opt^ was used as a negative control. Note that *Δc43* sporozoites were obtained from infections of *LRIM1* kd mosquitoes. Images are de-convoluted projection of confocal stacks. BF, bright field. (Scale bars, 5 μm.)

We used the α-Pfc43^opt^ antibody in indirect immunofluorescence assays to investigate the subcellular localization of PfPIMMS43 in *P. falciparum* NF54 parasite stages. Antibodies against the female gametocyte and ookinete surface protein Pfs25 and the sporozoite surface protein PfCSP (Circumsporozoite protein) were used as stage-specific controls. The results showed that PfPIMMS43 prominently localizes on the surface of female gametes or early-stage zygotes found in the *A. coluzzii* blood bolus 1 hpbf, as well as on the surface of ookinetes traversing the mosquito midgut epithelia and sporozoites found in the mosquito salivary gland lumen at 25 hpbf and 16 dpbf, respectively (Fig. 2*A*). There was no evidence of α-Pfc43^opt^ antibody staining of in vitro-cultured asexual blood stage or gametocytes, suggesting that expression of PfPIMMS43 protein starts after fertilization. No signal was detected with the α-Pfc43^opt^ rabbit preimmune serum that was used as a negative control.

Immunofluorescence assays of *P. berghei c507* and control *Δc43* parasite stages using the α-Pbc43^opt^ antibody revealed that, similarly to its *P. falciparum* ortholog, PbPIMMS43 localizes on the surface of *A. coluzzii* midgut-traversing ookinetes and salivary gland sporozoites (Fig. 2*B*). For the control *Δc43* line, which as reported below does not develop beyond the ookinete stage, sporozoites were obtained from infections of *LRIM1* knockdown (KD) mosquitoes (see below). Like PfPIMMS43, despite the presence of transcripts, no signal was detected in gametocytes. Furthermore, no PbPIMMS43 signal was detected in early-stage zygotes present in the blood bolus 1 hpbf, suggesting that translation starts later during ookinete development. In both species, the protein was detectable on the surface of 2-d-old oocysts found on the *A. coluzzii* midgut cell wall and reappeared in sporozoites found in mature *P. falciparum* oocysts 11 dpbf and *P. berghei* oocysts 15 dpbf (*SI Appendix*, Fig. S5).

### *PIMMS43* Deletion Mutants Fail to Reach Oocyst-Stage Infection.

**Fig. 3.**
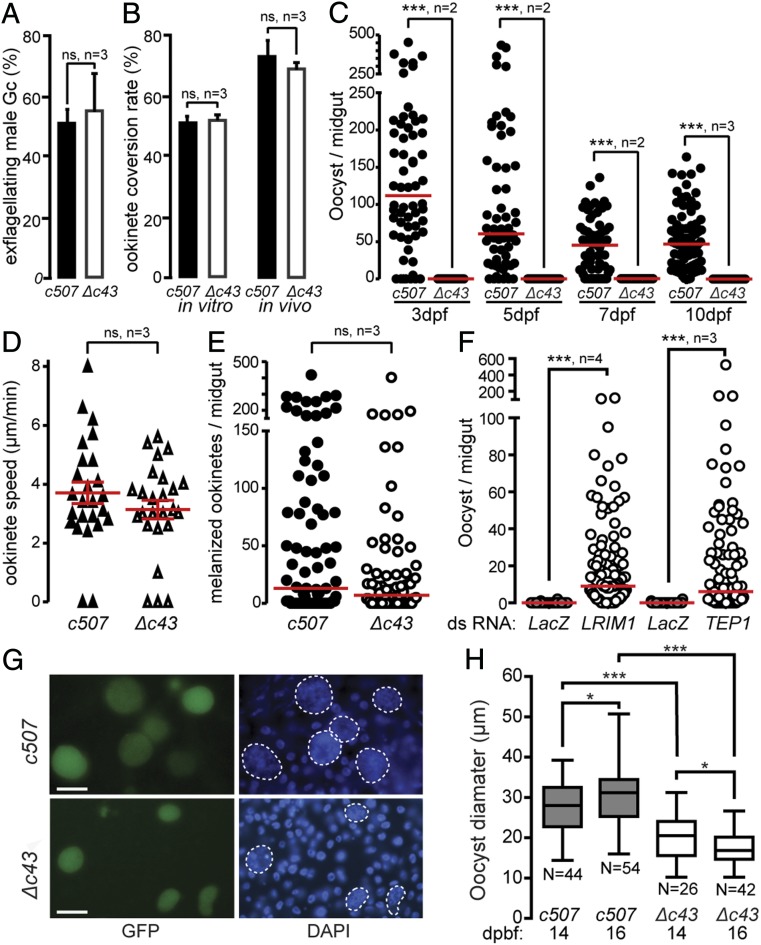
Phenotypic analysis of *P. berghei Δc43* KO mutant parasites. Male gametocyte activation measured as percentage of exflagellating male gametocytes (*A*) and female gamete conversion to ookinetes in vitro (*Left*), and in vivo in the *A. coluzzii* midgut of (*Right*) (*B*) of *c507 wt* and *Δc43* parasites. Error bars show SEM. (*C*) *Δc43* oocyst development at 3, 5, 7, and 10 dpbf in *A. coluzzii*. ***P < 0.0001, Mann–Whitney *U* test. (*D*) Speed of *c507 wt* and *Δc43* ookinetes measured from time-lapse microscopy, captured at one frame/5 s for 10 min. Red lines indicate mean and error bars show SEM. ns, not significant. (*E*) Melanized ookinete numbers in *CTL4* kd *A. coluzzii* infected with *c507 wt* and *Δc43* parasite lines. Red lines indicate median; ns, not significant; *n*, number of biological replicates. (*F*) Effect of *LRIM1* and *TEP1* silencing on *Δc43* oocyst numbers in *A. coluzzii* midguts. *dsLacZ*-injected mosquitoes were used as controls. Red lines indicate median; *n*, number of independent experiments; ***P < 0.0001, Mann–Whitney *U* test. (*G*) Representative images of rescued *Δc43* oocysts in *LRIM1* KD mosquitoes showing variable morphology and smaller size compared to *c507 wt* oocysts. (Scale bars, 30 µm.) (*H*) Box plot of diameter measurements of *Δc43* and *c507 wt* oocysts at 14 and 16 dpbf. Upper and lower whiskers represent the largest and smallest oocyst diameter, respectively. Horizontal line in each box indicates mean of 2 biological replicates and whiskers show SEM. *N* is number of oocysts; *P < 0.05, and ***P < 0.0001 using unpaired Student’s *t* test.

We phenotypically characterized the *P. berghei Δc43* line generated as described above. Consistent with the *PbPIMMS43* expression data, *Δc43* parasites exhibited normal development in mouse blood stages (*SI Appendix*, Fig. S6). Both male gametocyte activation, as measured by counting exflagellation centers (Fig. 3*A*), and macrogametocyte-to-ookinete conversion rate, both in vitro and in the *A. coluzzii* midgut lumen (Fig. 3*B*), were comparable to the *c507* parental line, indicating that no developmental defects are accompanying the parasite gametocyte-to-ookinete developmental transition. However, no oocysts were detected in *A. coluzzii* midguts at 3, 5, 7, or 10 dpbf, indicating complete abolishment of oocyst formation (Fig. 3*C* and *SI Appendix*, Table S1). Thus, oocyst and salivary gland sporozoites were never observed, and transmission to mice following mosquito bite-back was abolished (*SI Appendix*, Table S2).

To validate the specificity of this phenotype, we reintroduced *PbPIMMS43* into the *Δc43* locus by replacing the *TgDHFR* gene cassette with the *PbPIMMS43* coding sequence flanked by its 5′ and 3′ untranslated regions (UTRs) and followed by the human *DHFR* gene cassette (*SI Appendix*, Fig. S7*A*). Successful integration was confirmed with PCR (*SI Appendix*, Fig. S7*B*). Phenotypic characterization of the resulting *Δc43::c43*^*wt*^ parasite line in *A. coluzzii* infections showed that oocyst development was fully restored (*SI Appendix*, Fig. S7*C* and Table S1).

These data were in disagreement with those reported previously, which showed that *PSOP25* knockout (KO) parasites exhibit reduced ookinete conversion rates and defective ookinete maturation (21). To investigate this discrepancy, we generated a new *PIMMS43* KO (*Δc43*^*red*^) line in the *1804cl1* (*c1804*) *P. berghei* line that constitutively expresses mCHERRY (25), using the same disruption vector (PbGEM-042760) as the one used by the authors of the previous study, which leads to 74% removal of the gene coding region (*SI Appendix*, Fig. S8 *A* and *B*). Phenotypic analysis showed that *Δc43*^*red*^ parasites show normal ookinete conversion rates both in vitro and in *A. coluzzii* infections but produced no oocysts (*SI Appendix*, Fig. S8*C*), a phenotype identical to that of the *Δc43* line. Similar results were obtained in infections of *Anopheles stephensi*, the vector of choice in the previous studies (*SI Appendix*, Fig. S8*D*). Interestingly, the number of oocysts in *A. stephensi* infections was very small but not zero. This is consistent with the findings by Kaneko et al. (20), as well as with the general understanding that the *A. stephensi* Nijmegen strain, which was genetically selected for high susceptibility to parasite infections (26), has a less robust immune response than *A. coluzzii*. Nonetheless, no sporozoites were detected in the *A. stephensi* midgut 15 dpbf (*SI Appendix*, Fig. S8*E*).

### Ookinetes Lacking *PIMMS43* Are Killed by the Mosquito Complement-like Response upon Midgut Traversal.

We examined whether the *PIMMS43* KO phenotype was due to defective ookinete motility and, hence, capacity to invade or traverse the mosquito midgut epithelium. Ookinete motility assays showed that *Δc43* ookinetes moved on Matrigel with average speed that was not significantly different from *c507* ookinetes (Fig. 3*D*).

Next, a potential defect in midgut epithelium invasion and traversal was assessed in infections of *A. coluzzii* where *CTL4* (*C-type lectin 4*) was silenced by RNA interference. *CTL4* KD leads to melanization of ookinetes at the midgut subepithelial space upon epithelium traversal providing a powerful means to visualize and enumerate ookinetes that successfully traverse the midgut epithelium. The number of *Δc43* melanized ookinetes was comparable to that of the *c507* line that was used as control (Fig. 3*E* and *SI Appendix*, Table S3), indicating that *Δc43* ookinetes successfully traverse the midgut epithelium but fail to transform to oocysts.

A similar phenotype was previously reported for P47 KO parasites that are eliminated by mosquito complement-like responses upon emergence at the midgut subepithelial space (16). To examine whether the same applies to *Δc43* parasites, we infected *A. coluzzii* mosquitoes in which genes encoding two major components of the complement-like system, *TEP1* and *LRIM1*, were individually silenced. Enumeration of oocysts 10 dpbf, and comparison with control mosquitoes injected with *LacZ* double-stranded RNA, revealed that *Δc43* oocyst development was largely restored in both *TEP1* and *LRIM1* KD mosquitoes (Fig. 3*F*); although the numbers of recovered *Δc43* oocysts were still lower than the numbers of WT (*c507*) oocysts in *TEP1* and *LRIM1* KD mosquitoes (*SI Appendix*, Table S4).

Taken together, these data indicate that the absence of PIMMS43 does not affect the capacity of ookinetes to invade and traverse the mosquito midgut epithelium but instead required for protection from or evasion of the mosquito immune response. The observation that *Δc43* oocyst numbers are still inferior to WT parasite oocyst numbers in both *TEP1* and *LRIM1* KD mosquitoes could suggest that immune responses additional to TEP1-mediated killing of *Δc43* ookinetes. Indeed, it has been previously shown that some dead ookinetes in the midgut epithelium are not bound by TEP1 (7), indicating alternative means employed by the mosquito to kill *Plasmodium* ookinetes. Other mosquito immune factors, such as fibrinogen-related proteins (FREPs or FBNs) and LRRD7, are also important for midgut infection (27, 28). Of these, FBN9 is shown to colocalize with ookinetes in the midgut epithelium, probably mediating their death (28). Any such mechanism employed by the mosquito to kill *Δc43* ookinetes would have to be TEP1-independent. Since TEP1 attack is potentiated by prior marking of ookinetes by effector reactions of the JNK pathway (5, 6), it is plausible that *Δpbc43* ookinetes are excessively marked for death either by the same mechanism observed for *Pfs47*-null mutants or an independent mechanism. Alternatively, due to the spatial and temporal limitations of RNAi, it is possible that residual activity of TEP1 and LRIMI retained in the KD mosquitoes is responsible for the incomplete rescue of *Δc43* oocyst numbers. Nonetheless, all of the above scenarios suggest that PIMMS43, like P47, directly or indirectly interferes with the mosquito immune response promoting ookinete survival.

### PIMMS43 Is Additionally Required for Oocyst Maturation and Sporozoite Development.

We observed that the rescued *Δc43* oocysts in *LRIM1* or *TEP1* KD mosquitoes were morphologically variable and smaller in size compared to *c507* oocysts (Fig. 3*G*). At 14 and 16 dpbf the average *Δc43* oocyst diameter was 20.1 and 17.2 μm, compared to 27.4 and 30.9 μm of *c507* oocysts, respectively (Fig. 3*H*). All pairwise comparisons were statistically significant and revealed that the mean *Δc43* oocyst diameter at 16 dpbf was smaller than 14 dpbf, indicating progressive degeneration of *Δc43* oocysts. In addition, *Δc43* oocysts in *LRIM1* KD mosquitoes yielded a very small number of midgut and salivary gland sporozoites compared to *c507* oocysts, and the ratio of salivary gland to midgut sporozoites was significantly smaller for *Δc43* compared to control *c507* parasites (*SI Appendix*, Table S6). Furthermore, the few *Δc43* sporozoites that reached the salivary glands of in *LRIM1* KD mosquitoes could not be transmitted to mice through mosquito bites.

These data suggested that *Δc43* parasites are defective not only with respect to ookinete toleration of the mosquito complement-like response but also with sporozoite development and infectivity. We investigated whether bypassing midgut invasion, a process in which ookinetes are marked for elimination by complement-like reactions, could rescue *Δc43* sporozoite development and transmission to a new host. In vitro-produced *Δc43* and control *c507* ookinetes were injected into the hemocoel of *A. coluzzii* mosquitoes, and sporozoites found in the mosquito salivary glands 21 d later were enumerated. The results revealed that no *Δc43* sporozoites could be detected in the mosquito salivary glands, and consequently, mosquitoes inoculated with *Δc43* ookinetes could not transmit malaria to mice, in contrast to mosquitoes inoculated with *c507* ookinetes (*SI Appendix*, Table S6). While these data argue for an additional, essential function of PbPIMMS43 in sporozoite development, complement-independent effects directly impacting the injected ookinete may be responsible for the sporozoite phenotype observed in this experiment.

Next, we investigated whether PfPIMMS43 could complement the function of its *P. berghei* ortholog by infecting naïve *A. coluzzii* mosquitoes with the *Pb*^*Pfc43*^ parasite line and counting the number of oocysts detected in the mosquito midguts. Infections with *c507* and *Δc43* parasites served as positive and negative controls, respectively. The results showed that the *Pb*^*Pfc43*^ line exhibited an intermediate phenotype compared to *c507* and *Δc43* both in terms of both infection prevalence and intensity (*SI Appendix*, Fig. S4*E* and Table S4). Oocysts were morphologically variable and smaller in size compared to *c507* oocysts and contained a small number of sporozoites that could not be transmitted to a new mouse host (*SI Appendix*, Table S5), resembling the phenotype obtained with *Δc43* infections following silencing of the mosquito complement-like system. We examined whether this partial complementation phenotype could be affected upon *LRIM1* silencing. Indeed, a significant increase in both the infection prevalence and oocyst numbers was observed (*SI Appendix*, Fig. S4*E* and Table S4), yet oocysts remained small and morphologically variable and produced few sporozoites (*SI Appendix*, Table S5). These results suggest that PfPIMMS43 can only partly complement the function of its PbPIMMS43 ortholog and corroborate the dual function of PIMMS43 in ookinete to oocyst transition and in oocyst maturation and sporozoite development, respectively.

### *PIMMS43* KO Leads to Compromised Ookinete Fitness and Attack by the Complement-Like Response.

We carried out RNA next-generation sequencing of *P. berghei Δc43* and *c507* infected *A. coluzzii* midguts at 1 and 24 hpbf to further investigate the *Δc43* phenotype during mosquito midgut infection. *P. berghei* and *A. coluzzii* transcriptomes were processed separately, and comparatively analyzed at each time point for each parasite line (Datasets S1 and S2). Three independent biological replicates and three technical replicates for each biological replicate were performed.

**Fig. 4.**
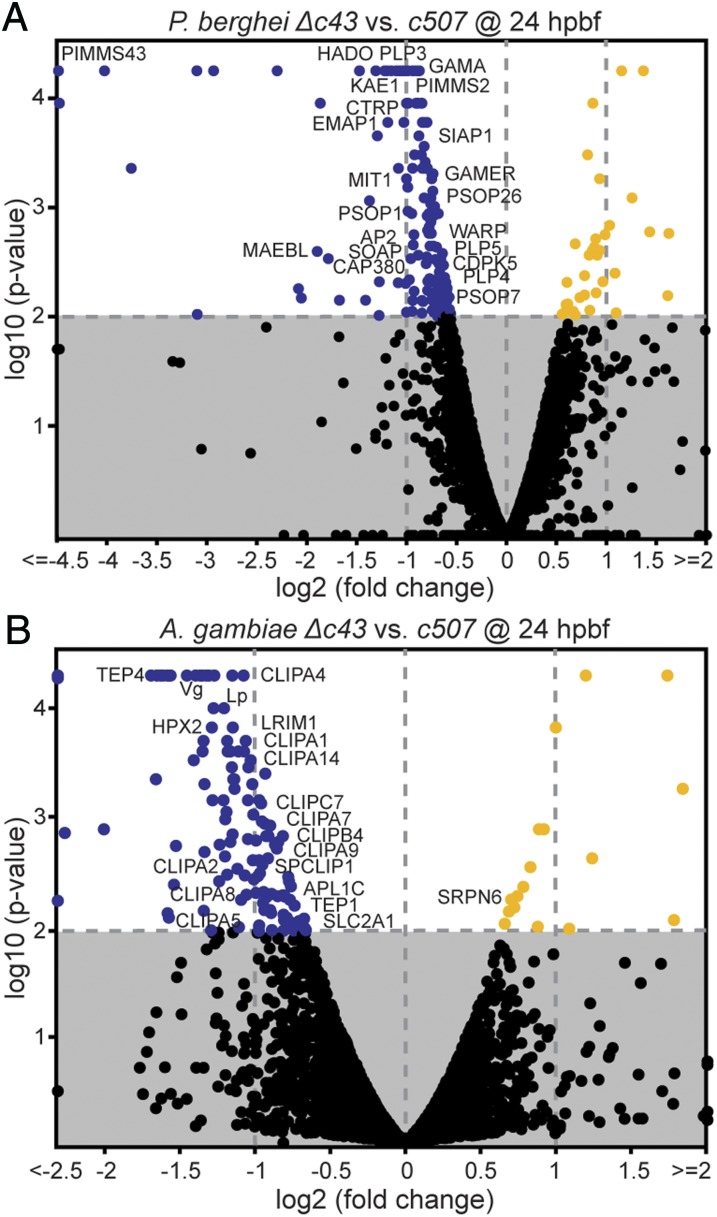
*P. berghei* and *A. coluzzii* gene expression 24 hpbf. (*A*) Volcano plot of *P. berghei* gene expression in *Δc43* vs. *c507 wt* parasite lines in the *A. coluzzii* midgut. (*B*) Volcano plots of *A. coluzzii* midgut transcriptional responses to *Δc43* vs. *c507 wt* parasites. The *x* axes show log_2_ fold-change and *y* axes show log_10_ *P* value calculated using one-way ANOVA. Blue and orange filled circles indicate genes that are at least twofold down down-regulated and twofold up-regulated, respectively. Black circles show with no significant differential regulation. Known gene names are indicated.

At 1 hpbf, when asexual parasite stages and gametocytes are sampled from the mosquito blood bolus, almost all 17 changes registered between *Δc43* and *c507* parasites concerned genes belonging to multigene families (*pir*, *fam-a*, and *fam-b*) and ribosomal RNAs (*SI Appendix*, Fig. S9*A*). We hypothesize that this is due to differential expression of such genes between clonal parasite lines rather than differences related to the disruption of *PbPIMMS43*. As many as 163 genes were differentially regulated between the *Δc43* and *c507* parasites at 24 hpbf, of which 137 were down-regulated (41 at least twofold) and 26 were up-regulated (9 at least twofold) (Fig. 4*A*). Gene ontology analysis revealed several biological processes and three cellular component terms that were significantly enriched in the differentially regulated gene set (*SI Appendix*, Table S7). All gene ontology terms were related to host–parasite interactions, including micronemal secretion, entry into host cell, and parasite movement. Genes included in this list encode known ookinete secreted or membrane-associated proteins—such as CTRP, SOAP, MAEBL, WARP, PLP3-5, PIMMS2, HADO, PSOP1, PSOP7, PSOP26, GAMA (also known as PSOP9)—and others, all of which were down-regulated in *Δc43* parasites. The expression of the oocyst capsule protein *Cap380* gene that begins in ookinetes was also affected (29).

These data could be explained by a smaller ratio of ookinetes to other parasite stages sampled from the midgut at 24 hpbf in *Δc43* infections compared to *c507* infections. Although the data from the ookinete melanization assays showed that differences between *Δc43* and *c507* in ookinete numbers exiting the mosquito midgut were not statistically significant (*P* = 0.0947), these differences were almost twofold both with regards to median and arithmetic mean (*SI Appendix*, Table S3). This difference could justify the observed twofold down-regulation of genes showing enriched expression in ookinetes. A second hypothesis is that *Δc43* parasites exhibit deficient expression of genes involved in ookinete secretions and movement. The latter hypothesis is less appealing, as it is difficult to explain how absence of a membrane-associated protein without obvious signaling domains could affect the transcription of all other genes. However, the two hypotheses are not mutually exclusive, and both indicate that disruption of *PIMMS43* leads to compromised ookinete fitness.

Analysis of *A. coluzzii* midgut transcriptional responses to infection by *Δc43* compared to *c507* identified 192 and 122 differentially regulated genes at 1 and 24 hpbf, respectively (Dataset S2). At 1 hpbf, 154 (88 over twofold) genes were down-regulated and 38 (21 over twofold) were up-regulated (*SI Appendix*, Fig. S9*B*). However, these genes did not appear to follow any functional pattern, and annotation enrichment analyses did not yield any significant results. In contrast, at 24 hpbf, and although the number of identified genes was smaller (109 down-regulated, 71 over twofold; 13 up-regulated, 5 over twofold), most genes shown to date to be involved in systemic immune responses of the complement-like system and downstream effector reactions—including *TEP1*, *LRIM1*, *APL1C*, and various clip-domain serine protease homologs—were down-regulated (Fig. 4*B*). Enrichment analysis confirmed that the serine protease/protease/hydrolase and the serine protease inhibitor/protease inhibitor protein classes were significantly overrepresented in this gene list. When considered together with the increased complement activity observed against *Δc43* compared to the *c507* ookinetes, these data could suggest induction of a negative feedback mechanism to down-regulate this self-damaging innate immune response. However, most of these genes are thought to be largely, and in some cases exclusively, expressed in hemocytes and fat body cells; therefore, their detection as down-regulated in midgut tissues cannot be easily explained. Thus, a more possible explanation is that midgut infection by *Δc43* ookinetes causes mobilization and differentiation of hemocytes attached to the midgut tissues, as shown previously (30–32), causing a temporal depletion of relevant transcripts from the midgut tissue.

We examined this hypothesis by measuring the abundance of transcripts encoding the three major components of the complement-like system, TEP1, LRIM1, and APL1C, in the midgut and whole body (excluding legs, wings, and heads) of *A. coluzzii* mosquitoes infected with *Δc43* or control *c507* parasites at 24 hpbf. Since the *Δc43* phenotype was similar to the *Δpbp47* phenotype (16), and because preliminary data indicated similar *A. coluzzii* midgut responses to the two mutant parasite lines, transcript abundance in infections with *Δpbp47* parasites were also examined. The results revealed a striking difference in transcript abundance of all three genes between midgut and whole mosquitoes (*SI Appendix*, Fig. S10). In accordance with the RNA-sequencing data, the relative transcript abundance in infections with the two mutant parasite lines compared to control infections was lower in the midgut but higher in whole mosquitoes. These data are consistent with our hypothesis that ookinetes lacking PIMMS43 or P47 trigger hemocyte mobilization and consequent depletion in the midgut tissue, although the up-regulation of the three genes in whole mosquitoes could also be explained by up-regulation in other immune tissues, such as the fat body.

### *PIMMS43* Exhibits Significant Geographic Structure Among African *P. falciparum* Populations.

It has been shown that Pfs47 presents strong geographic structure in natural *P. falciparum* populations, both between continents and across Africa (33–35). Furthermore, a small-scale genotypic analysis of oocysts sampled from *A. gambiae* and *Anopheles funestus* mosquitoes in Tanzania revealed significant differentiation in Pfs47 haplotypes sampled from the two vectors (36). These data are consistent with natural selection of Pfs47 haplotypes by the mosquito immune system and a key role of this interaction in parasite–mosquito coevolution (34). However, a different study showed that polymorphisms in the *Pfs47* locus alone could not fully explain the observed variation in complement-mediated immune evasion of African *P. falciparum* strains (37).

**Fig. 5.**
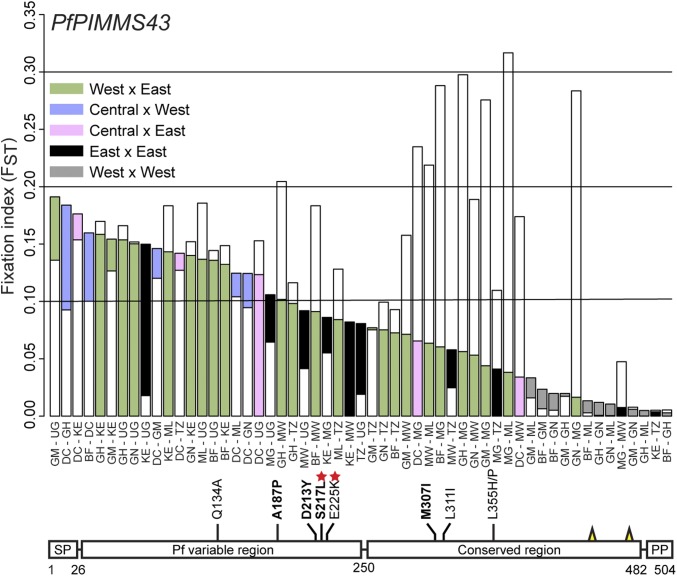
Population genetic analysis of *PfPIMMS43* in African *P. falciparum*. *PfPIMMS43 F*_*ST*_ values of 1,509 *P. falciparum* populations sampled from patients across Africa (*Upper*) and schematic representation of SNPs with high *F*_*ST*_ values leading to amino acid substitutions in each deduced protein (*Lower*). (*Upper*) Color coding indicates comparisons between countries in West, Central, and East Africa. Central Africa includes populations sampled only from the DC. White bars overlaid with colored bars indicate the *F*_*ST*_ of *Pfs47*. (*Lower*) Boldfaced amino acid substitutions are those deriving from SNPs with total *F*_*ST*_ > 0.1, and the rest of the substitutions are those showing high *F*_*ST*_ in comparison between populations sampled from specific countries. Substitutions marked with red stars are those showing very high *F*_*ST*_ and have swept to almost fixation in some populations. Yellow spikes show the positions of conserved cysteine residues. Burkina Faso, BF; Democratic Republic of the Congo, DC; Gambia, GM; Ghana, GH; Guinea, GN; Kenya, KE; Madagascar, MG; Malawi, MW; Mali, ML; Tanzania, TZ; Uganda, UG.

We investigated the genetic structure of African *P. falciparum* populations with regards to *PfPIMMS43*, and compared this to the structure of *Pfs47*, using a rich dataset of 1,509 genome sequences of parasites sampled from 11 African countries in the context of the *P. falciparum* Community Project (http://www.malariagen.net/). The *PfPIMMS43* analysis revealed significant population differentiation as determined by the Fixation Index (*F*_*ST*_), mostly between populations of some West or Central (Democratic Republic of the Congo, DC) and East African countries (*F*_*ST*_ > 0.1) (Fig. 5). The highest *F*_*ST*_ is detected in comparisons of Ugandan, DC, or Kenyan populations with West African populations. The most differentiated single nucleotide polymorphisms (SNPs) are detected within the nonconserved region that is unique to *P. falciparum* (Dataset S3). Within this region, an SNP that leads to the nonsynonymous substitution of Serine-217 to Leucine (S217L) is highly differentiated between sampled Kenyan/Tanzanian and all other populations, while a nearby SNP that leads to substitution of Glutamate-226 to Lysine (E225K) has swept to almost fixation in Ugandan populations.

The *PIMMS43 F*_*ST*_ profile does not fully match the *F*_*ST*_ profile of *Pfs47* that also presents strong genetic differentiation between West and East Africa but is particularly strong for populations sampled in Madagascar and Malawi versus West African and DC populations (*SI Appendix*, Fig. S11). The most highly differentiated SNPs are within domain 2 (D2) of the protein (Dataset S3). An SNP leading to substitution of Leucine-240 to Isoleucine (L240I) is almost fixed in Madagascar and Ugandan versus West African populations, while a nearby SNP leading to the nonsynonymous substitution of Asparagine-271 to Isoleucine (N271I) is highly prevalent in DC versus all other populations, especially those sampled from East Africa. Our analysis also detected all four SNPs previously shown to differentiate between African (NF54) and New World (GB8) *P. falciparum* laboratory lines and lead to amino acid substitutions in the D2 region that contribute to immune evasion (38); however, these SNPs were neither highly prevalent nor did they present significant geographic structure apart from that leading to Isoleucine-248 substitution to Leucine or Valine (I258L/V) that is significantly prevalent (*F*_*ST*_ > 0.1) in sampled Ugandan populations. These data concur with the hypothesis presented previously that polymorphisms in the D2 region of Pfs47, even those leading to synonymous substitutions, can alter the parasite immune evasion properties (38). Finally, one of the substitutions defining the East versus West African differentiation is that of Glutamate-27 to Aspartate (E27D) at the start of the mature protein. This SNP is almost fixed in sampled Madagascar populations.

Together, these data reveal that *PfPIMMS43* and *Pfs47* exhibit significant geographic structure, consistent with their deduced role in parasite immune evasion. They also suggest that different selection pressures are exerted on each of these genes, which concurs with the hypothesis that the two proteins serve different functions. A major difference between West and East African vector species is the presence of both *A. gambiae* (*A. gambiae* S-form) and *A. coluzzii* (*A. gambiae* M-form) in West Africa but only *A. gambiae* in East Africa. Interestingly, a resistant allele of *TEP1*, *TEP1r*^*B*^, is shown to have swept to almost fixation in West African *A. coluzzii* but be absent from *A. coluzzii* sampled from Cameroon, consistent with the high *PfPIMMS43 F*_*ST*_ observed between Central and West African parasite populations, as well as from all sampled *A. gambiae* populations (39). Therefore, it is tempting to speculate that a difference between West and East African vectors in their capacity to clear parasite infections through complement responses may have contributed to the observed *PfPIMMS43* and *Pfs47* genetic structure.

Moreover, *A. funestus* and *A. arabiensis* appear to have recently taken over from *A. gambiae* as the primary malaria vectors in many areas of East Africa (40), in contrast to West Africa where *A. gambiae* and *A. coluzzii* remain the primary vectors. While nothing is known about the capacity of *A. funestus* to mount complement-like responses against malaria parasites, *A. arabiensis* is shown to be a less good vector of *P. berghei* but can be transformed into a highly susceptible vector, equal to *A. gambiae*, when its complement system is silenced (41). Finally, *Anopheles merus* is only found in coastal East Africa; although its abundance and contribution to malaria transmission has been increasing (42) it is unlikely that it has majorly contributed to structuring parasite populations.

### Incubation with Anti-PIMMS43 Antibodies Significantly Reduces Malaria Transmission by *A. coluzzii*.

**Fig. 6.**
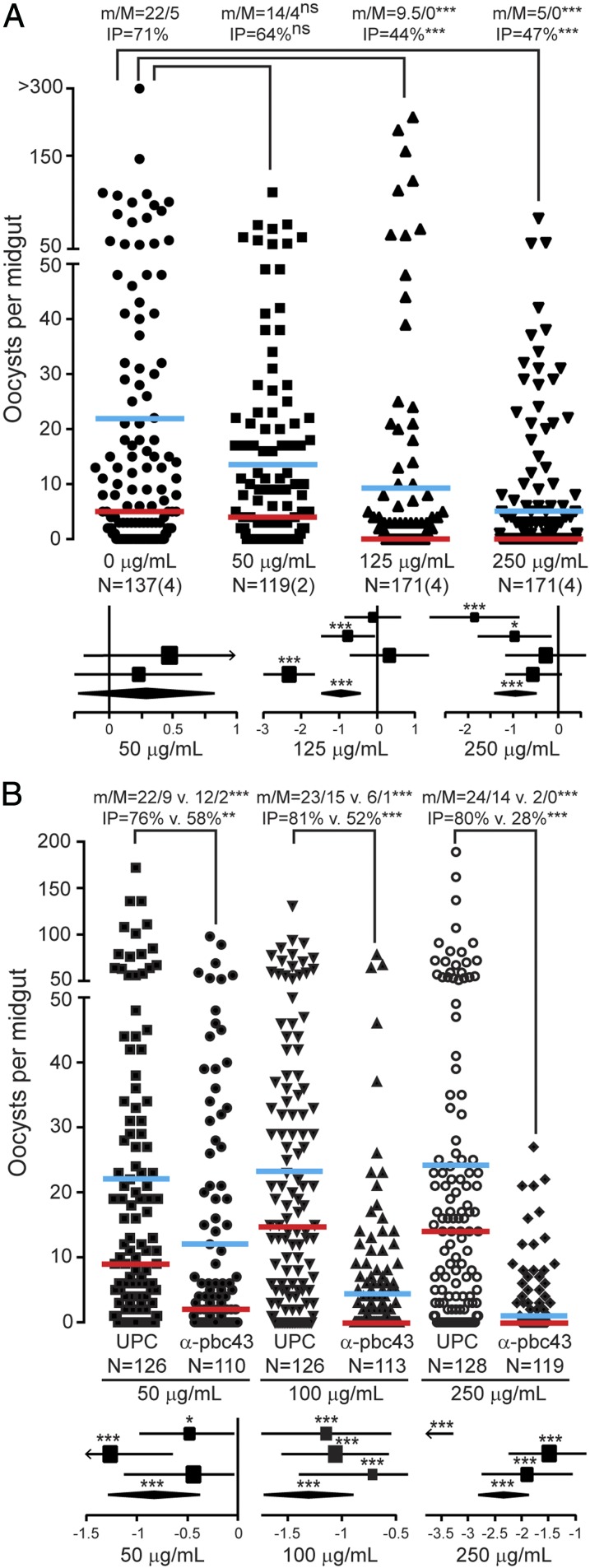
*P. falciparum* and *P. berghei* transmission blocking with anti-PIMMS43 antibodies. Transmission-blocking efficacies of anti-PIMMS43 antibodies on (*A*) *P. falciparum* and (*B*) *P. berghei* infections of *A. coluzzii* shown as dot plots of oocyst number distribution (*Upper*) and forest plots of generalized linear mixed-model analysis (*Lower*). The α-Pfc43^opt^ and α-Pbc43^Sf9^ antibodies were provided through SMFAs at concentrations of 50, 125, and 250 µg/mL, and 50, 100, and 250 µg/mL, respectively, and compared with no antibodies and UPC10 antibodies that were used as negative controls for *P. falciparum* and *P. berghei*, respectively. Individual data points represent oocyst numbers from individual mosquitoes at 7 and 10 dpbf from two/four and three biological SMFA replicates with *P. falciparum* and *P. berghei*, respectively. m/M are mean/median oocyst infection intensities, also shown as horizontal blue and red lines, respectively. IP, oocyst infection prevalence; *N*, number of midguts analyzed; *n*, number of independent experiments; ns, not significant. Statistical analysis was performed with Mann–Whitney *U* test for infection intensity and Fisher’s exact test for infection prevalence; **P < 0.005; ***P < 0.0001. In generalized linear mixed-model analyses, the variation of fixed-effect estimates for each replicate (squares) and all replicates (diamonds) are shown (±95% confidence interval, glmmADMB). The square size is proportional to the sum of midguts analyzed in each replicate. *P < 0.05; ***P < 0.0001.

We examined in both *P. falciparum* and *P. berghei* whether targeting PIMMS43 using antibodies generated against each of the respective orthologous proteins could reduce parasite infectivity and malaria transmission potential. For *P. falciparum* transmission-blocking assays, purified IgG α-Pfc43^opt^ antibodies were added to gametocytemic blood at final concentrations of 0, 50, 125, and 250 μg/mL prior to offering this as bloodmeal to female *A. coluzzii* mosquitoes through optimized standard membrane feeding assays (SMFAs) (43). Oocysts present in mosquito midguts at day 7 postfeeding were enumerated. The results showed strong inhibition of both infection intensity and infection prevalence in an antibody dose-dependent manner (Fig. 6*A* and *SI Appendix*, Table S8). At 125 and 250 μg/mL of antibody following four biological replicates, the overall inhibition of infection intensity observed was 57.1% and 76.2%, and the overall inhibition of infection prevalence was 37.3% and 35.6%, respectively (*P* < 0.0001).

Similar results were obtained with *P. berghei* transmission upon addition of α-Pbc43^Sf9^ antibodies to blood drawn from infected mice and provided to mosquitoes as bloodmeal in SMFAs. Statistically significant inhibition of both infection intensity and prevalence was detected at all antibody concentrations tested (i.e., 50, 100, and 250 μg/mL) in an antibody dose-dependent manner (Fig. 6*B* and *SI Appendix*, Table S9). At 100 μg/mL, the inhibition of oocyst intensity was 72.7% and the inhibition of infection prevalence was 35.5%, and these values increased to 90.3% and 65.6% at 250 μg/mL, respectively (*P* < 0.0001).

A recent study has shown that antibodies binding a 52-amino acid region of Pfs47 confer strong transmission blocking of laboratory *P. falciparum* strains in *A. gambiae* (44). In the same study, antibodies binding different regions of the protein showed either weak or no transmission-blocking activity, consistent with an earlier study reporting that none of three monoclonal antibodies against Pfs47 could affect *P. falciparum* infections in *A. stephensi* (45). These findings agree with the general understanding that antibodies binding different regions of a targeted protein can have profound differences in their blocking activity, especially when antibodies have a primarily neutralizing function (46, 47). Indeed, our polyclonal α-Pbc43^opt^ antibody raised against codon-optimized PbPIMMS43 expressed in *E. coli* cells appeared to not exhibit any transmission-blocking activity against *P. berghei*, despite producing strong signals in Western blot analyses and immunofluorescence assays. However, antibodies against fragments of PSOP25 (synonym of PIMMS43) expressed in *E. coli* cells have been previously shown to inhibit *P. berghei* infection in *A. stephensi* (21, 48), albeit not as strongly as our α-Pbc43^Sf9^ antibodies.

### Concluding Remarks and Perspectives.

We demonstrate that PIMMS43 is required for parasite evasion of the mosquito immune response, a role also shared by P47 in both *P. falciparum* and *P. berghei* (14, 16). The mechanism by which these molecules exert their function is unclear. A general explanation may lie with their GPI constituents or with their structural role in the formation of the ookinete sheath. On the one hand, *Plasmodium* GPIs are known to modulate the vertebrate host immune system (49), and studies have shown that mosquitoes mount a specific immune response against GPIs (50, 51). On the other hand, the integrity of the ookinete sheath may be important for counteracting attacks by or acting as molecular sinks of free radicals produced during traversal of midgut epithelial cells (5, 6) or by directly enduring the attack of the complement-like system. Ookinetes lacking such membrane proteins may thus be irreversibly damaged and subsequently eliminated by the mosquito complement-like response. In relation to this, a specific function could be attributed to the conserved cysteine residues present in these proteins. Apart from their role in forming disulphide bridges, thus serving a structural purpose, the ability of cysteine thiol groups to regulate the redox potential may be relevant (52). Interestingly, midgut infection with *P. berghei* is shown to inhibit the expression of catalase that mediates the removal of free radicals, and silencing catalase exacerbates ookinete elimination (53). Nonetheless, population genetic analyses indicate a more specific role of the two proteins in parasite–mosquito interactions and coadaptation.

Notwithstanding their exact function in parasite immune evasion, PIMMS43, P47, and possibly other proteins involved in parasite immune evasion are targets of interventions aiming to block malaria transmission in the mosquito. One such approach is transmission-blocking vaccines aiming at generating antibodies in the human serum which, when ingested by mosquitoes together with gametocytes, interfere with the function of these proteins and block transmission to a new host (54). Several putative transmission-blocking vaccines are currently being investigated at a preclinical stage, including those targeting the gametocyte and ookinete proteins Pfs230, Pfs48/45, and Pfs25 (55). Another, more ambitious approach is the generation of genetically modified mosquitoes expressing single-chain antibodies or nanobodies that bind these proteins conferring refractoriness to infection and leading to malaria transmission blocking (56, 57). Such genetic features can be spread within wild mosquito populations in a super-Mendelian fashion via means of gene drive (e.g., CRISPR/Cas9) and can lead to sustainable local malaria elimination (58).

## Materials and Methods

### Ethics Statement.

All animal procedures were reviewed and approved by the Imperial College Animal Welfare and Ethical Review Body and carried out in accordance with the Animal Scientifics Procedures Act 1986 under the United Kingdom Home Office licenses PLL70/7185 and PPL70/8788. Human red blood cells provided by the National Blood Service of the United Kingdom National Health Service were obtained from healthy donors upon taking a written informed consent.

### Protein Expression and Purification.

Recombinant *PfPIMMS43* and *PbPIMMS43* were expressed in *E. coli* (*PfPIMMS43* and *PbPIMMS43*) and *S. frugiperda* insect cells (*PbPIMMS43*). Fragments without the signal peptide and the C-terminal hydrophobic domain were expressed as Histidine-fusion proteins. Details of the cloning, expression, and purification are found in *SI Appendix*, *Supplementary Materials and Methods*.

### Generation of Transgenic Parasites.

Two transgenic parasites carrying 50% and 74% KO of the *P. berghei PIMMS43* coding DNA sequence were created by homologous recombination in the *P. berghei c507* and *1804cl1* lines respectively. For the generation of *P. berghei* transgenic parasites expressing *P. falciparum PIMMS43*, the *PfPIMMS43* coding sequence was expressed under the control of the *PbPIMMS43* 5′UTR and 3′UTR and at the original *PbPIMMS43* locus in the *P. berghei c507* line. For the complementation assays, we reintroduced the full length *PbPIMMS43* in the Δc43 line under the control of its 5′UTR and 3′UTR and at its original locus. Details of the creation of these transgenic parasites are found in *SI Appendix*, *Supplementary Materials and Methods*.

### RNA-Sequencing.

Total RNA was isolated from midguts infected with WT and *PbPIMMS43* KO parasites at 1 and 24 hpbf. The RNA was used to prepare cDNA libraries (New England Biolabs Ultra prep kit) and run on an Illumina HiSEq. 3000 instrument. For details, see *SI Appendix*, *Supplementary Materials and Methods*.

### Transmission-Blocking Assays.

Polyclonal antibodies generated against the above recombinant proteins were utilized in transmission-blocking assays. Here, mosquitoes were fed with *P. berghei* and *P. falciparum* by SMFAs in the presence of the generated polyclonal antibodies. Oocyst infection intensity and oocyst infection prevalence was determined at 10 dpbf. See *SI Appendix*, *Supplementary Materials and Methods*.

### Other Materials and Methods.

Materials and methods for RT-PCR and qRT-PCR, Western blot analysis, immunofluorescence assays, phenotypic assays, and population genetic analysis are described in detail in in *SI Appendix*, *Supplementary Materials and Methods*.

### Data Availability Statement.

The RNA-sequencing data are available through the European Nucleotide Archive with experiment codes ERX3197375 to ERX3197410.

## Supplementary Material

Supplementary File

Supplementary File

Supplementary File

Supplementary File
