# Supplementary material for: PIMMS43 is required for malaria parasite immune evasion and sporogonic development in the mosquito vector
Source: Proc Natl Acad Sci U S A. 2020 Mar 12;117(13):7363–73. doi: 10.1073/pnas.1919709117 (PMC7132314; doi:10.1073/pnas.1919709117)
Supplement: Supplementary File [file pnas.1919709117.sapp.pdf]

Supplementary Information for

PIMMS43 is required for malaria parasite immune evasion and sporogonic development in the mosquito vector

Chiamaka V. Ukegbu, Maria Giorgalli, Sofia Tapanelli, Luisa D.P. Rona, Amie Jaye, Claudia Wyer, Fiona Angrisano, Andrew M. Blagborough, George K. Christophides, Dina Vlachou

George K. Christophides, Dina Vlachou
Emails: g.christophides@imperial.ac.uk, d.vlachou@imperial.ac.uk

This PDF file includes:
Figures S1 to S11
Tables S1 to S10
Supplementary Materials and methods
Legends for Datasets S1 to S3
SI References

Other supplementary materials for this manuscript include the following:
Datasets S1 to S3

www.pnas.org/cgi/doi/10.1073/pnas.1919709117

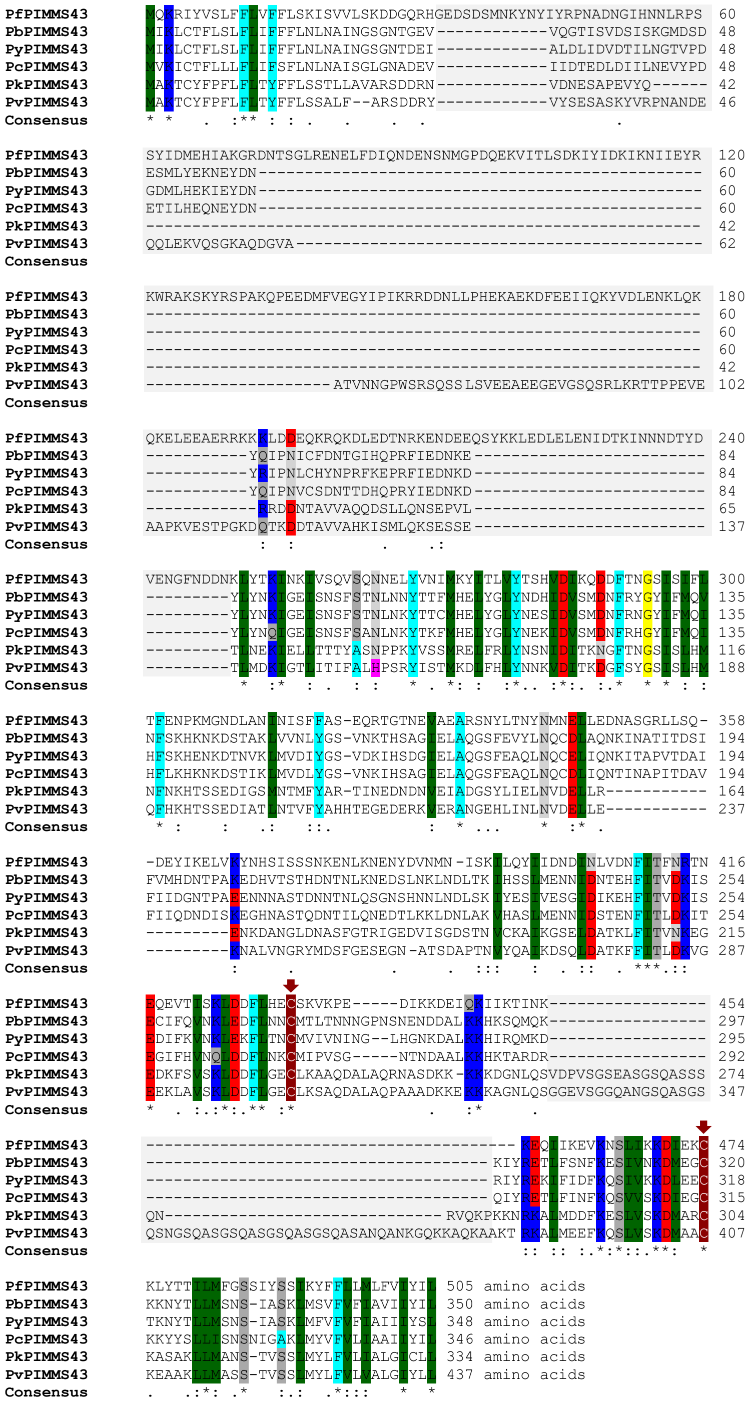

Figure S1. Multiple sequence alignment of *Plasmodium* PIMMS43 orthologs

Sequence alignment of PIMMS43 orthologs in six *Plasmodium* species: *P. falciparum* (PfPIMMS43; PF3D7_0620000), *P. berghei* (PbPIMMS43; PBANKA_1119200), *P. yoelii* (PyPIMMS43; PYYM_1121200), *P. chabaudi* (PcPIMMS43; PCHAS_1118700), *P. knowlesi* (PkPIMMS43; PKNH_1130300) and *P. vivax* (PvPIMMS43; PVX_114125). Amino acid residues are color-shaded according to their biochemical characteristics. Conserved Cysteine residues are indicated with arrows. The two variable regions are highlighted with grey background color. In the consensus sequence, dots and colons mark conserved amino acid residues with weakly and strongly similar properties, respectively, and asterisks mark amino acid residues that are identical between orthologs. Protein sequences were retrieved from PlasmoDB.

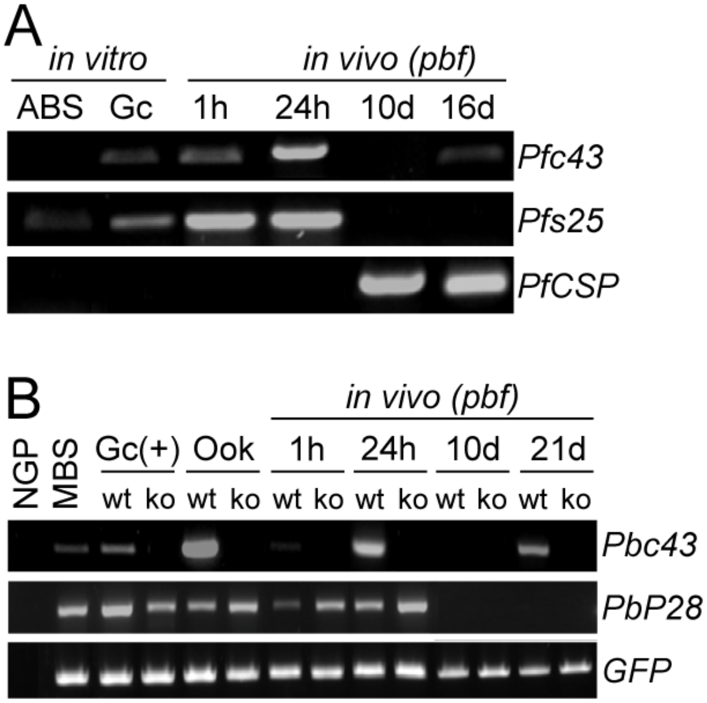

Figure S2. PIMMS43 RT-PCR transcription profiles

(A) RT-PCR analysis of *Pfc43* transcripts in *in vitro* and *in vivo* NF54 parasite populations. Gametocyte expressed gene *Pfs25* and sporozoite-expressed gene *PfCSP* serve as both stage-specific and loading controls. **(B)** RT-PCR analysis of the expression of *Pbc43* transcripts in blood stages, *in vitro* ookinetes and *A. coluzzii* mosquito stages. Gametocyte expressed gene *P28* and constitutive expressed *GFP* served as a stage-specific and loading controls, respectively. *Δc43* parasites were used as a negative control. Abbreviations: ABS, asexual blood stages; NGP, non-gametocyte producing; MBS, mixed blood stages; Gc, gametocytes; Gc (+), activated gametocytes; Ook, ookinetes; pbf, post blood feeding; wt, wild-type (c507); ko, *Δc43* knockout.

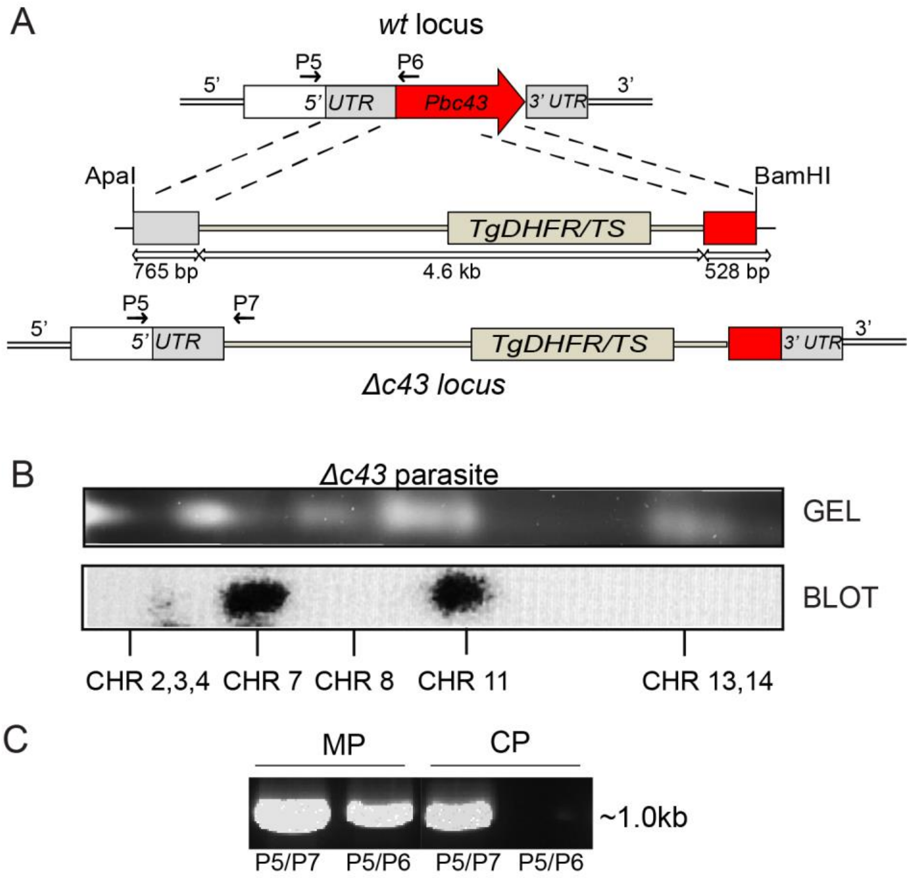

Figure S3. Generation of *P. berghei Δc43* knock out mutant in the *c507* reference line
(**A**) Schematic representation of *Pbc43* locus disruption by double crossover homologous recombination. The disruption vector design allows for 50% ko of the coding DNA sequence. Arrows indicate binding sites for primers P5, P6 and P7 used in diagnostic PCR. P5 and P7 were used to detect integration and P5 and P6 that bind to the endogenous *PbPIMMS43* locus were used to confirm absence of the endogenous gene in the ko line. (**B-C**) Genotypic analysis of *Δc43* mutants following transfection and dilution cloning by (**B**) southern blot analysis on pulsed field gel electrophoresis separated transgenic chromosomes and (**C**) PCR on mix parasite (MP) and *Δc43* clonal populations (CP). Used primer combinations are shown below each detected band.

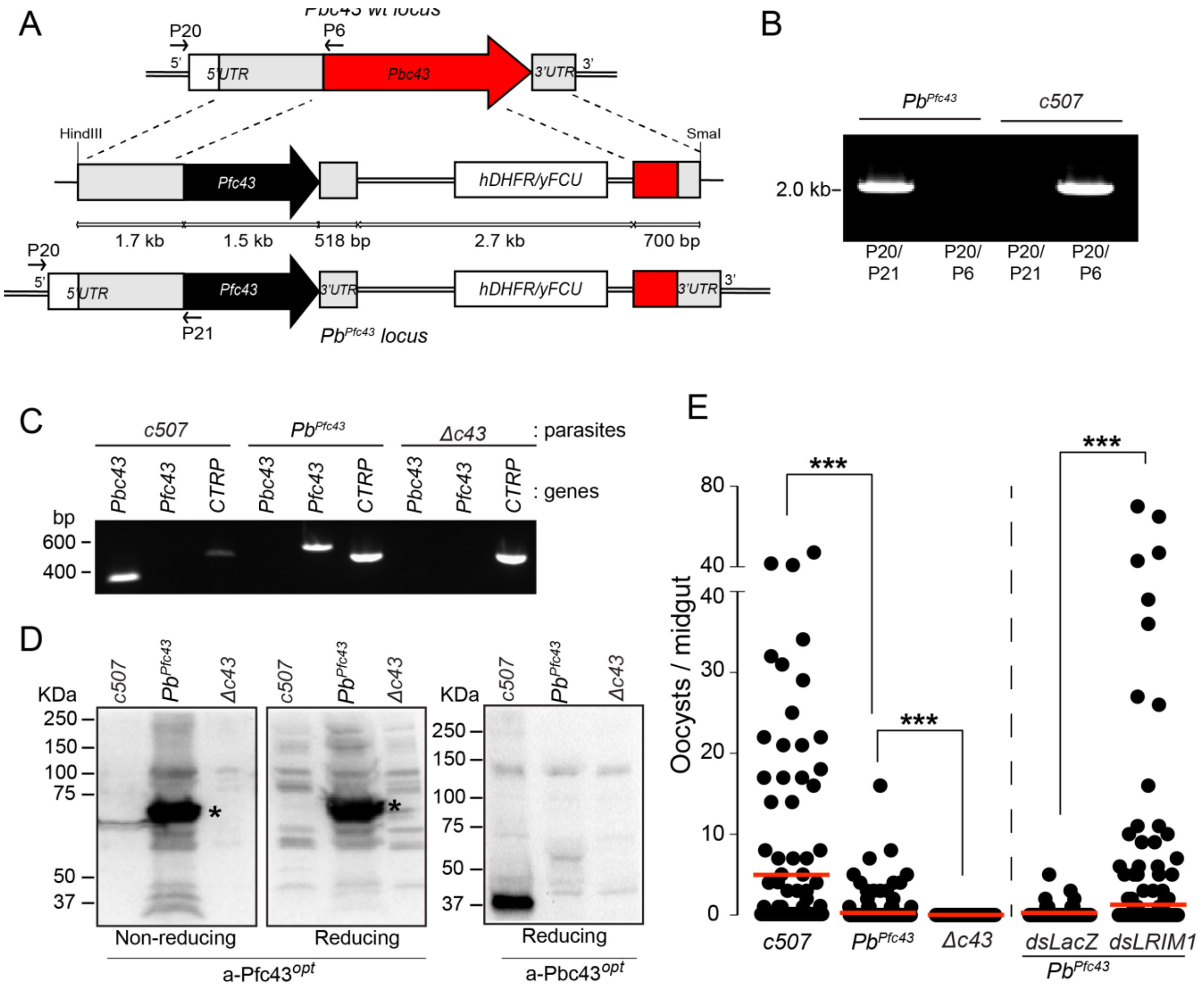

Figure S4. Generation and phenotypic analysis of *Pb*^{Pfc43}

(**A**) Schematic diagram of the endogenous *Pbc43* locus, targeting construct and resulting transgenic *Pb*^{Pfc43} locus. Arrows P6, P20 and P21 indicate binding sites for primers used in diagnostic PCR. P20 and P21 were used to detect integration, and P20 and P6 that bind to the endogenous *Pbc43* locus were used to confirm absence of the endogenous gene in the transgenic line. (**B)** Diagnostic PCR to determine integration of the *Pfc43* targeting construct into the endogenous *Pbc43* locus. The *c507 wt* parasite served as a control. (**C**) RT-PCR in ookinetes to confirm expression of *Pfc43* in the *Pb*^{Pfc43} transgenic line. Amplifications of *Pbc43* and *CTRP* were used as positive control for the endogenous locus and stage-specific control, respectively. (**D**) Western blot analysis under reducing and no-reducing conditions of whole *c507*, Δc43 and *Pb*^{Pfc43} parasite cell lysates using the α-Pbc43^{opt} and α-Pfc43^{opt} antibodies. Pfc43 protein bands are indicated with asterisks. (**E**) Oocyst density in naïve and *LRIM1* kd *A. coluzzii* infected with the *Pb*^{Pfc43} transgenic line. The *c507 wt* and *Δc43* parasites served as controls in naïve mosquito infections. Red horizontal lines indicate median. ***, P<0.0001 with Mann-Whitney test.

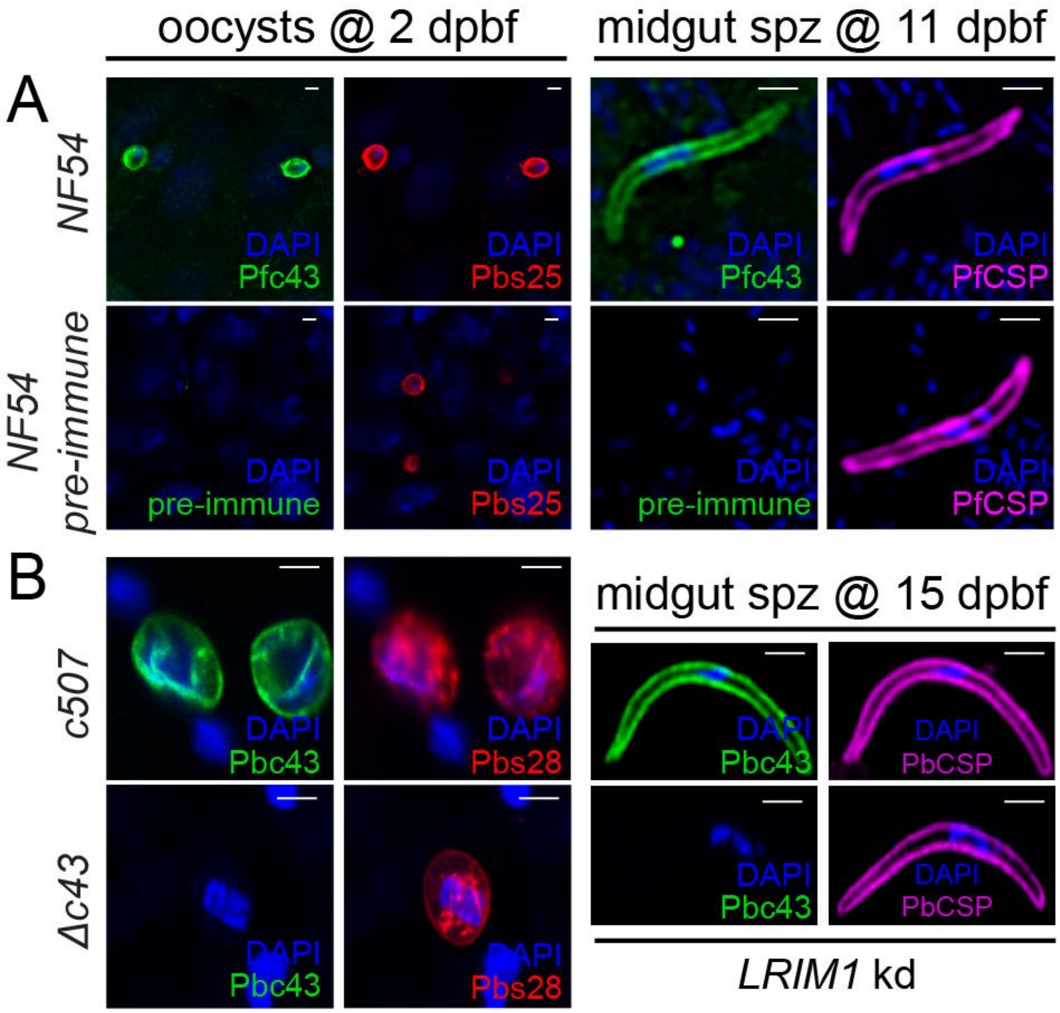

Figure S5. PIMMS43 localization on the surface of young oocysts and midgut sporozoites in *A. coluzzii* infected midgut epithelia

(A) Immunofluorescence assays of NF54 *P. falciparum* oocysts found in the mosquito midgut epithelium at 2 dpbf (left) and midgut/oocyst sporozoites (spz) at 11 dpbf (right), stained with α-Pfc43^{opt} (green), α-Pfs25 (red) and/or α-PfCSP (purple) antibodies. DNA was stained with DAPI. Staining with pre-immune serum was used as a negative control. **(B)** Immunofluorescence assays of *P. berghei c507* parasites found in the mosquito midgut epithelium at 2 dpbf (left) and oocyst sporozoites at 15 dpbf (right), stained with α-Pbc43^{opt} antibody (green), α-P28 (red) and/or α-PbCSP (purple) antibodies. DNA was stained with DAPI. Staining with pre-immune serum was used as a negative control. Images in both panels are de-convoluted projection of confocal stacks. BF denotes bright field and scale bars correspond to 5 μm.

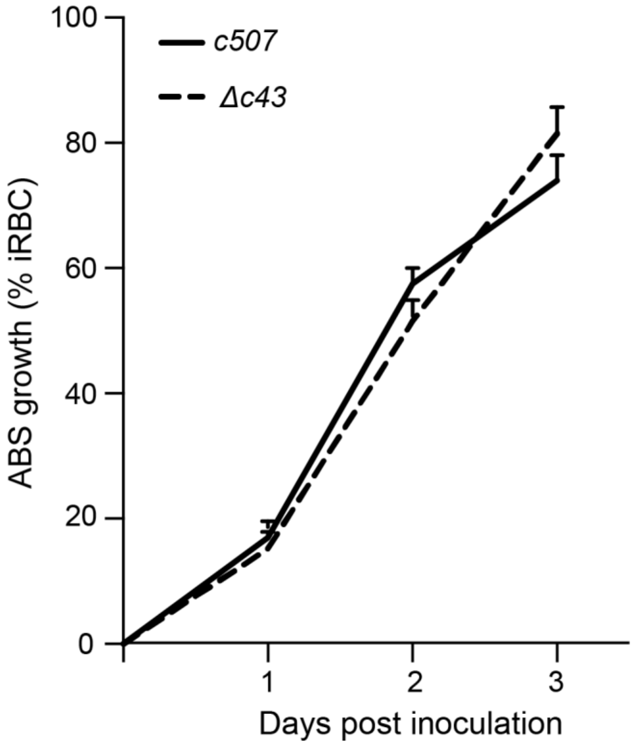

Figure S6. Asexual blood stage (ABS) growth of *Δc43* and control *c507* parasites
ABS growth is measured every 24 hours upon parasite inoculation for 3 consecutive days and expressed as percentage (%) of infected red blood cells (iRBC). The arithmetic mean of two biological replicates and the standard error of the mean (SEM) are shown.

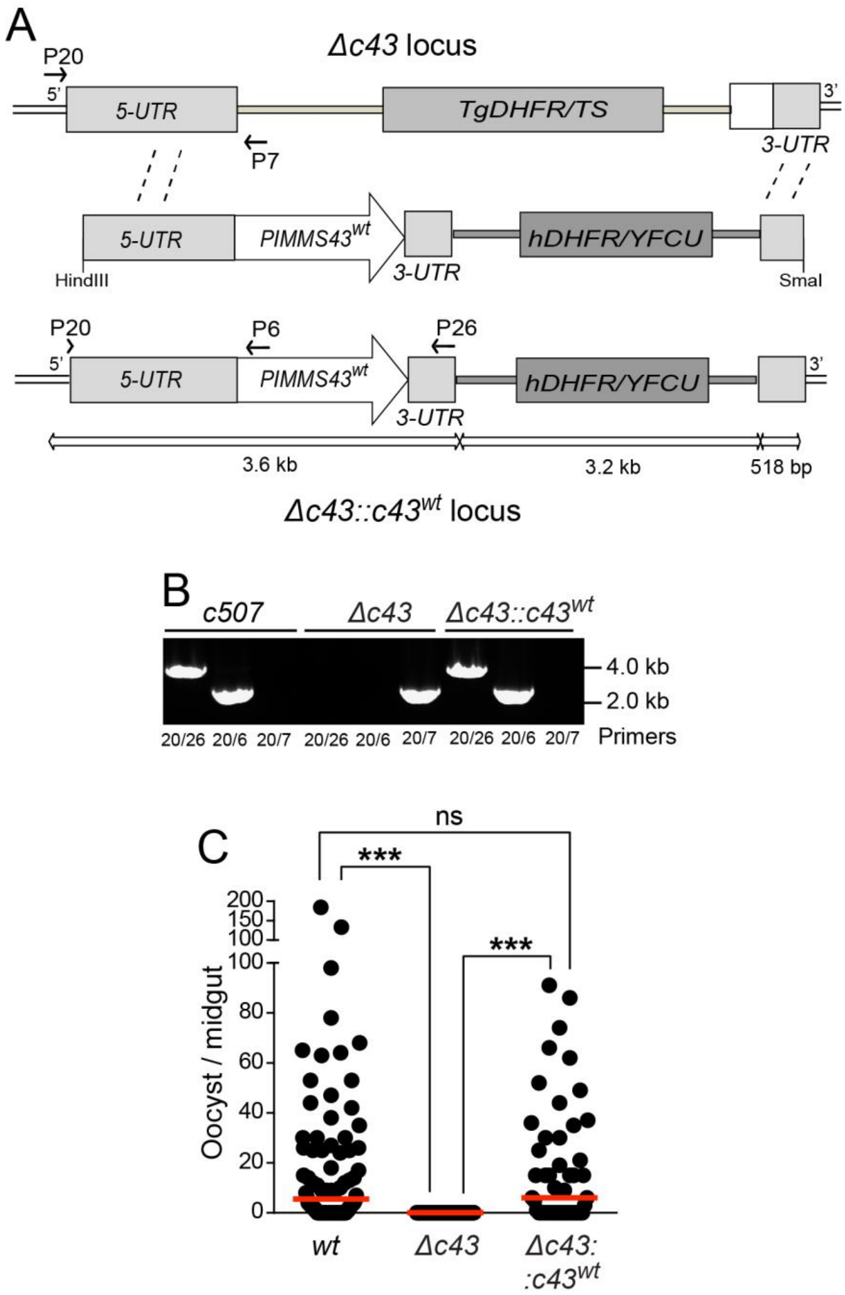

Figure S7. Complementation of *Δc43* mutant with endogenous *PbPIMMS43 wt* allele

(A) Schematic diagram of the *Δc43* ko locus, targeting construct and transgenic *Δc43::c43*^{wt} complemented locus. Arrows indicate binding sites of primers used in diagnostic PCR. P20, P6 and P26 detect integration and are used to confirm re-introduction of the *wt Pbc43* in the *Δc43::c43*^{wt} transgenic line. P20 and P7 bind to the *Δc43* ko locus. **(B)** Diagnostic PCR to determine integration of the targeting construct into the *Δc43* ko locus. The *c507 wt* and *Δc43* parasites served as PCR controls. **(C)** Oocyst density in *A. coluzzii* infected with the *Δc43::c43*^{wt} transgenic line. The *c507 wt* and *Δc43* parasites served as controls. Red horizontal lines indicate the median; ns, not significant; ***, P<0.0001 with Mann-Whitney test.

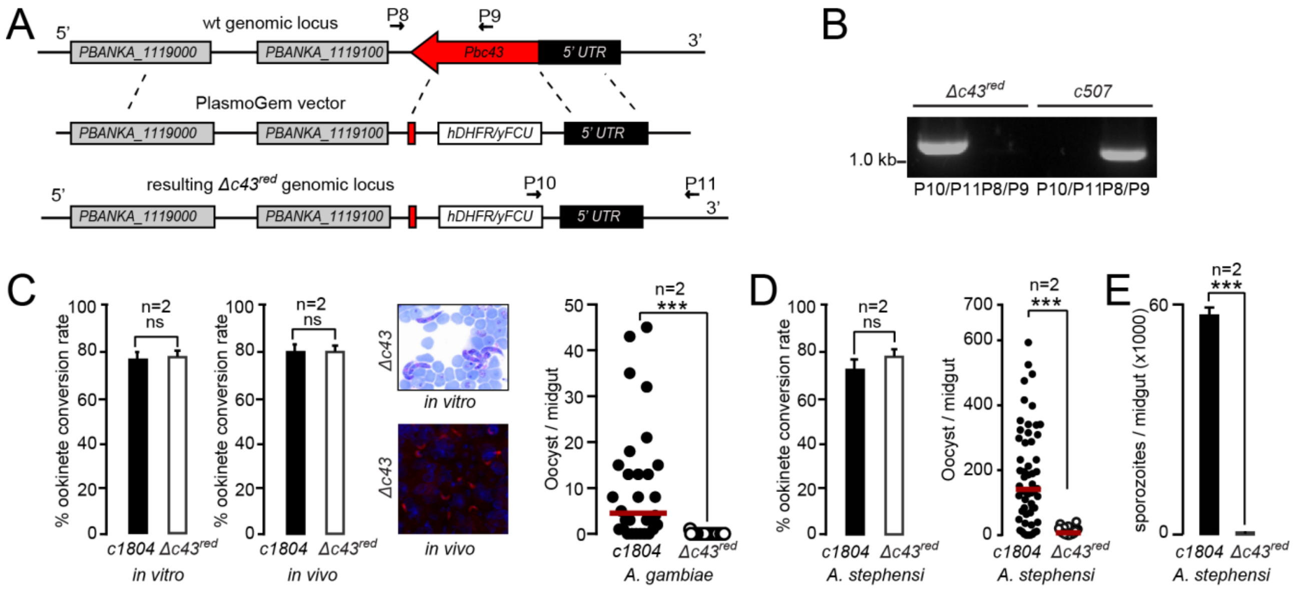

Figure S8. Generation and phenotypic analysis of *P. berghei Δc43^{red}* mutant line

(A) Schematic representation of *PbPIMMS43* (*Pbc43*) disruption using the PlasmoGEM vector, PbGEM: 042760. The design allows for 74% removal of the gene coding sequence. Arrows indicate binding sites of primers P8-11 used in diagnostic PCR assays. P10 and P11 were used to detect integration, and P8 and P9 bind to the endogenous locus and were used to confirm absence of the endogenous gene in the ko parasite. **(B)** Diagnostic PCR to determine integration of the targeting construct into the endogenous locus. (**C**) Female gametocyte to ookinete conversion rate *in vitro* (left), and *in vivo* in the *A. coluzzii* midgut (middle left) of *P. berghe*i *c1804* and mutant *Δc43^{red}* lines. Error bars indicate SEM. Representative images of *in vivo* invading and *in vitro* produced *Δc43^{red}* ookinetes are shown (middle right). The graph on the right shows numbers of *c1804* and mutant *Δc43^{red}* oocysts in the midguts of *A. coluzzii* mosquitoes 10 dpbf. **(D)** Gametocyte to ookinete conversion rate in the midgut bolus (left) and oocyst numbers (right) of *c1804* and *Δc43^{red} P. berghei* lines in *A. stephensi* mosquitoes. (**E**) Sporozoite numbers of *c1804* and *Δc43^{red} P. berghei* lines in the midgut of *A. stephensi* mosquitoes. In all the graphs, red lines indicate median, n is the number of independent experiments, ns and *** denote non-significant P values and P<0.0001, respectively, assessed with the Mann-Whitney test.

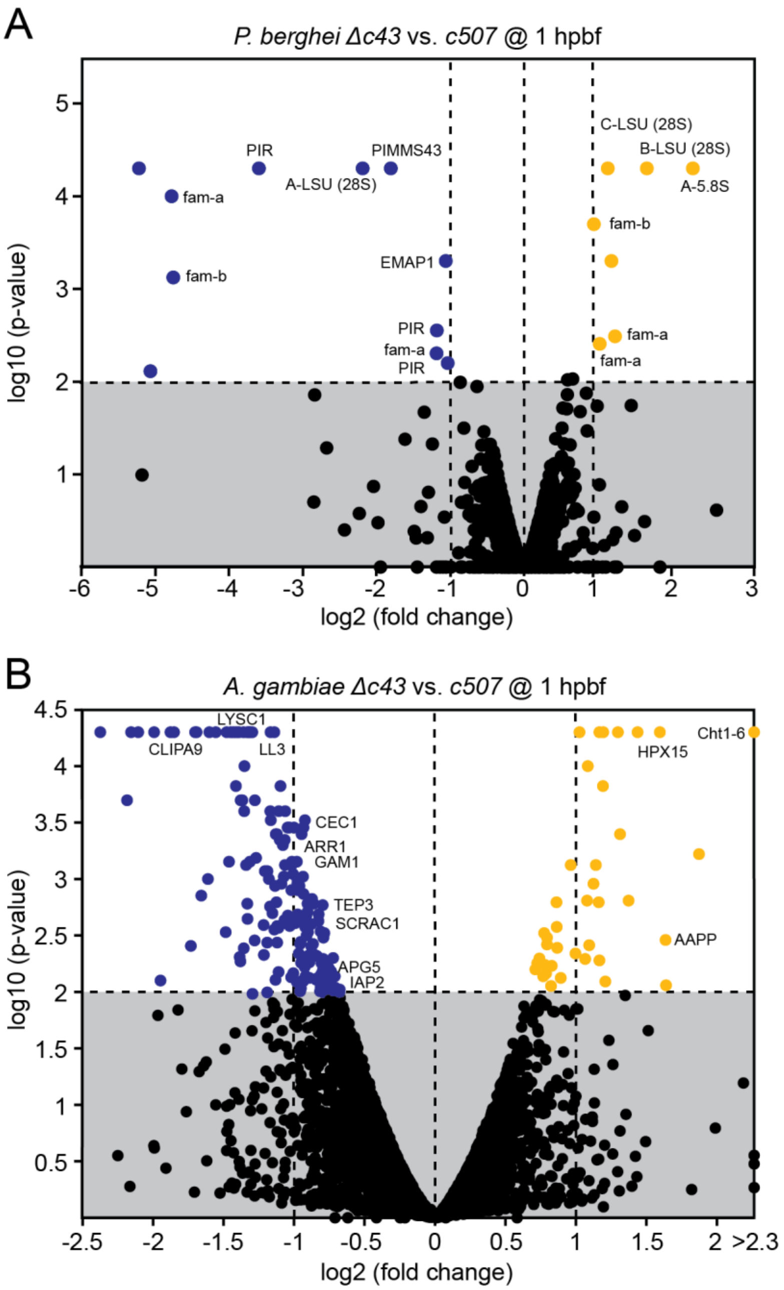

Figure S9. *P. berghei* and *A. coluzzii* gene expression 1 hpbf
(**A**) Volcano plot of *P. berghei* gene expression in *Δc43* vs. *c507 wt* parasite lines in the *A. coluzzii* midgut. (**B**) Volcano plot of *A. coluzzii* midgut transcriptional responses to *Δc43* vs. *c507 wt* parasites. X-axes show log_2 fold change and y-axes show log_{10} p-value calculated using one-way ANOVA. Blue and orange filled circles indicate genes that are at least 2-fold down downregulated and 2-fold upregulated, respectively. Black circles show with no significant differential regulation. Known gene names are indicated.

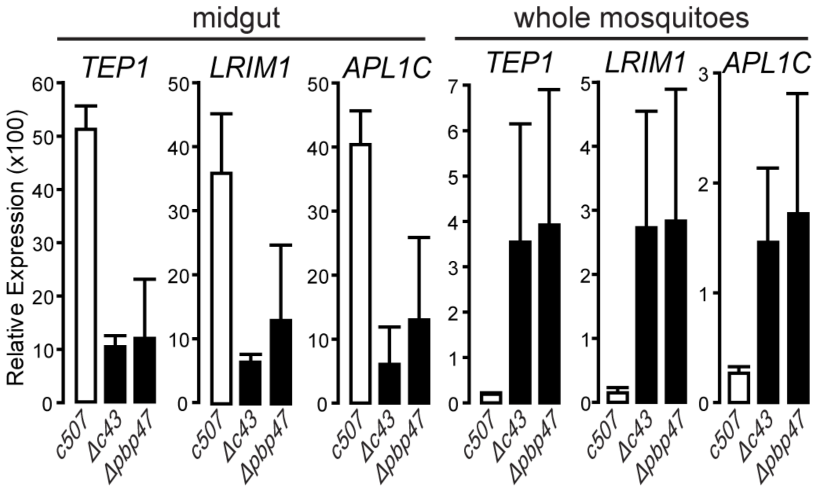

Figure S10. Transcriptional profiles of TEP1, LRIM1 and APL1C in infected *A. coluzzii* tissues

Relative abundance of *TEP1*, *LRIM1* and *APL1C* transcripts in the midgut and whole body of *A. coluzzii* mosquitoes infected with the *c507*, *Δc43* or *Δpbp47* parasite lines, measured by qRT-PCR at 24 hpbf. Whole body refers to mosquito tissues after the removal of wings, legs and head. Data are derived from two independent replicates, normalized to the abundance of mosquito *S7* transcripts in each of the two tissues and referenced to data obtained at 1 hpbf that was used as baseline. Error bars indicate SEM.

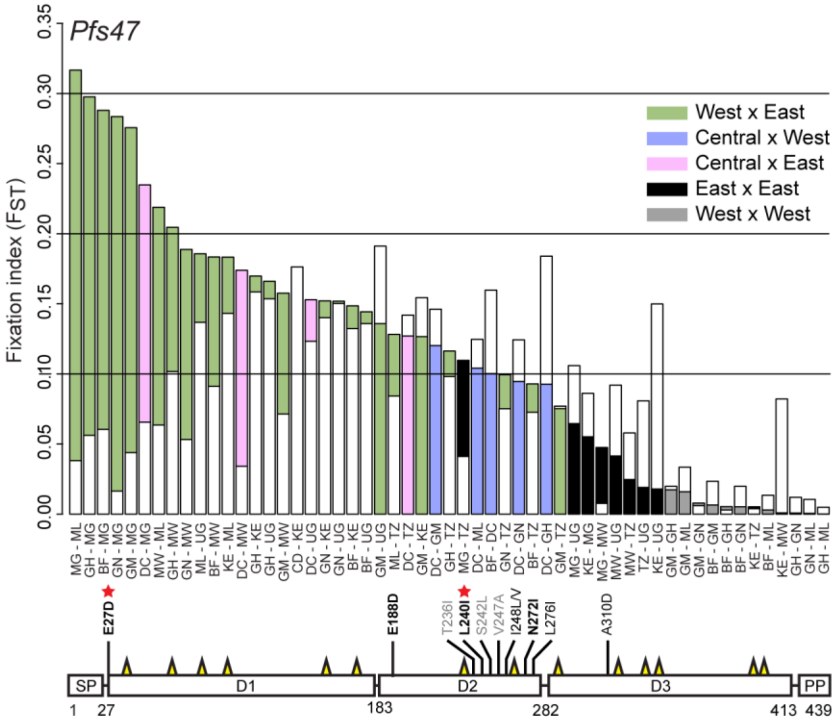

Figure S11. Population genetic analysis of *Pfs47* in African *P. falciparum*

Pfs47 fixation index (F_{ST}) values of 1,509 *P. falciparum* populations sampled from patients across Africa (top panel) and schematic representation of SNPs with high F_{ST} values leading to amino acid substitutions in each deduced protein (bottom panel). In top panel, colour coding indicates comparisons between countries in West, Central and East Africa. Central Africa includes populations sampled only from the Democratic Republic of the Congo. White bars overlaid with coloured bars indicate the F_{ST} of *PfPIMMS43*. In bottom panel, boldfaced amino acid substitutions are those deriving from SNPs with total F_{ST}>0.1, and the rest of the substitutions are those showing high F_{ST} in comparisons between populations sampled from specific countries. Substitutions presented in grey do not show high F_{ST} but have been shown previously to be present in laboratory NF54 *P. falciparum* and be involved in parasite immune evasion. Substitutions marked with red stars are those showing very high F_{ST} and have swept to almost fixation in some populations. Yellow spikes show the positions of conserved Cysteine residues. Burkina Faso, BF; Democratic Republic of the Congo, DC; Gambia, GM; Ghana, GH; Guinea, GN; Kenya, KE; Madagascar, MG; Malawi, MW; Mali, ML; Tanzania, TZ; Uganda, UG.

.

Table S1. Oocyst numbers in *A. coluzzii* infections

Dataset	Days post infection	Parasite	Number of experiments	Number of midguts	Prevalence (%)	Arithmetic mean	Median	Parasite range	*P* value
I (Figure 3C)	3dpi	*c507*	Pool	60	88	134.6	112	0-453	
		Δc43		63	0	0	0	0	<0.0001
		c507	R1	27	93	132.4	99	0-378	
		Δc43		33	0	0	0	0	<0.0001
		c507	R2	33	85	136.5	123	0-453	
		Δc43		30	0	0	0	0	<0.0001
	5dpi	*c507*	Pool	62	90	97.7	61	0-435	
		Δc43		58	0	0	0	0	<0.0001
		Δc43	R1	26	0	0	0	0	
		c507		29	93	85.2	59	0-362	<0.0001
		Δc43	R2	32	0	0	0	0	
		c507		62	90	97.7	61	0-435	<0.0001
	7dpi	*c507*	Pool	63	78	44.6	45	0-136	
		Δc43		56	0	0	0	0	<0.0001
		c507	R1	32	75	41.9	34	0-136	
		Δc43		26	0	0	0	0	<0.0001
		c507	R2	31	81	47.4	47	0-104	
		Δc43		30	0	0	0	0	<0.0001
	10dpi	*c507*	Pool	86	98	52.6	47	0-164	
		Δc43		86	0	0	0	0	<0.0001
		c507	R1	30	100	38.6	26	8-164	
		Δc43		30	0	0	0	0	<0.0001
		c507	R2	24	92	63.4	63	0-142	
		Δc43		24	0	0	0	0	<0.0001
		c507	R3	32	100	57.6	57	1-149	
		Δc43		32	0	0	0	0	<0.0001
II (Figure S5D)	10dpi	*c507*	Pool	84	69	19	6	0-184	
		*Δc43::c43^wt^*		49	59	18	6	0-91	0.8826
		Δc43		51	0	0	0	0	<0.0001
		c507	R1	42	62	18.6	6	0-133	
		*Δc43::c43^wt^*		25	60	17.8	10	0-86	0.7279
		Δc43		27	0	0	0	0	<0.0001
	10dpi	*c507*	R2	42	76	18.8	5	0-184	
		*Δc43::c43^wt^*		24	58	18.5	4	0-91	0.5823
		Δc43		24	0	0	0	0	<0.0001

Oocyst data in the midgut of *A. coluzzii* mosquitoes infected with the *c507*, *Δc43* or *Δc43::c43^{wt} P. berghei* lines. Data from pooled and independent biological replicates are presented. *P* values were calculated using Mann-Whitney test. Datasets I and II are independent of each other.

Table S2. Sporozoite numbers of *Δc43* and *c507* parasites in *A. coluzzii* infections

Parasite	Replicates	Midgut sporozoites		Salivary gland sporozoites		Infectivity to mice
		Mean	SEM	Mean	SEM	Day 21
c507	Pool	2,249	473	2,146	235	9 of 9
Δc43		0	0	0	0	0 of 9
c507	R1	1,990 (2,100/1,850/2,020)	60	2,715 (2,982/2,447)	189	3 of 3
Δc43		0	0	0	0	0 of 3
c507	R2	1,400 (1,650/1,118/1,430)	126	1,788 (2,361/1,215)	405	3 of 3
Δc43		0	0	0	0	0 of 3
c507	R3	3,358 (2,504/4,070/3,500)	374	1,936 (1,830/2,041)	75	3of 3
Δc43		0	0	0	0	0 of 3

Mean oocyst and salivary gland sporozoite numbers from three biological replicates of *A. coluzzii* infections with *Δc43* or *c507* parasite lines. For each biological replicate, sporozoite numbers was determined from two or three pools (technical replicates) of ten homogenized mosquito midguts or salivary glands at 15 and 21 days post infection respectively. Data from pooled and independent biological replicates are presented. The data from each technical replicate are also presented in brackets. Infectivity of sporozoites was assessed by infected mosquito bite back experiments with at least 30 mosquitoes on C57/BL6 mice at 21 dpi. Following this, parasitaemia was monitored until 14 days post mosquito bite. SEM represents standard error of mean.

Table S3. Invasion assay in *CTL4* knockdown *A. coluzzii*

Parasite	Number of experiments	Number of midguts	Prevalence (%)	Arithmetic mean	Median	Parasite range	*P* value
c507	Pool	83	65	62.1	13	0-418	
Δc43		79	62	30.3	7	0-403	0.0947
wt	R1	35	46	27.0	2	0-107	
Δpbc43		35	54	22.9	4	0-194	0.929
wt	R2	32	75	71.4	45	0-281	
Δpbc43		26	73	18.5	8	0-136	0.0543
wt	R3	16	69	120.4	80	0-418	
Δpbc43		18	61	61.6	14	0-403	0.1917

Numbers of melanised parasites detected in the midguts of *c507* or *Δc43* infected *A. coluzzii* mosquitoes at 4 days post infection. The *p* value was calculated using the Mann-Whitney U test. Data from pooled and independent biological replicates are presented.

Table S4. Effect of *A. coluzzii* gene silencing on oocyst numbers

Dataset	Parasite	Mosquito	Number of experiments	Number of midguts	Prevalence (%)	Arithmetic mean	Median	Parasite range	*P* value
I	*Δc43*	*LacZ*	Pool	132	2	0.03	0	0-2	
(Figure 3F)		*LRIM1*		128	82	17.2	9	0-119	<0.0001
		LacZ	R1	40	8	0.1	0	0-2	
		LRIM1		32	100	24	15	2-111	<0.0001
		LacZ	R2	32	0	0	0	0	
		LRIM1		39	74	18.50	10	0-119	<0.0001
		LacZ	R3	20	0	0	0	0	
		LRIM1		22	68	13.41	4	0-95	<0.001
		LacZ	R4	40	0	0	0	0	
		LRIM1		35	83	12.26	5	0-80	<0.0001
	Δc43	*LacZ*	Pool	104	3	0.03	0	0-2	
		TEP1		102	76	23.4	6	0-525	<0.0001
		LacZ	R1	40	8	0.1	0	0-2	
		TEP1		37	86	28.03	8	0-525	<0.0001
		LacZ	R2	32	0	0	0	0	
		TEP1		35	66	27.01	6	0-146	<0.0001
		LacZ	R3	32	0	0	0	0	
		TEP1		30	77	13.50	3	0-96	<0.0001
	c507	*LacZ*	Pool	49	88	16.9	11	0-88	
		LRIM1		45	89	176.8	140	0-676	<0.0001
		TEP1		23	96	71.1	56	0-286	<0.0001
		LacZ	R1	21	90	16.76	11	0-56	
		LRIM1		24	96	192	190	0-676	<0.0001
		TEP1		8	100	54.38	58	16-96	<0.005
		LacZ	R2	28	86	17.07	11	0-88	
		LRIM1		21	81	159.48	98	0-491	<0.005
II	*c507*	Naive	Pool	54	73	10.3	4	0-47	
(Figure S3E)	Pb^{Pfc43}	Naive		79	20	0.8	0	0-16	<0.0001
	Δc43	Naive		63	0	0	0	0	
	c507	Naive	R1	42	64	8.8	2.5	0-47	
	Pb^{Pfc43}	Naive		67	16	0.6	0	0-16	<0.0001
	Δc43	Naive		43	0	0	0	0	
	c507	Naive	R2	12	92	15.5	15.5	0-32	
	Pb^{Pfc43}	Naive		11	46	2.1	0	0-8	<0.0001
	Δc43	Naive		20	0	0	0	0	
III	Pb^{Pfc43}	*LacZ*	Pool	77	13	0	0	0-5	

(Figure S3E)	*Pb*^{Pfc43}	*LRIM1*		71	55	9	1	0-70	<0.0001
	Pb^{Pfc43}	*LacZ*	R1	24	12.5	0.125	0	0-1	
	Pb^{Pfc43}	*LRIM1*		23	70	10	3	0-70	<0.0001
	Pb^{Pfc43}	*LacZ*	R2	25	8	0.12	0	0-2	
	Pb^{Pfc43}	*LRIM1*		18	50	8.5	1	0-45	0.0003
	Pb^{Pfc43}	*LacZ*	R3	28	18	0.42	0	0-5	
	Pb^{Pfc43}	*LRIM1*		30	47	4.1	0	0-36	0.008

Numbers of *Δc43*, c507 or *Pb*^{Pfc43} oocysts in the midguts of naïve, *LacZ* or LRIM1 or TEP1 dsRNA injected *A. coluzzii* mosquitoes at 10 days post blood feeding on infected mice. *P* values were calculated using the Mann-Whitney U test. Datasets I, II and III are independent of each other. Data from pooled and independent replicates are presented.

Table S5. Sporozoite numbers in *LRIM1* silenced *A. coluzzii* infections

		Oocyst sporozoites		Salivary gland sporozoites		
Parasite	Knockdown	Mean	SEM	Mean	SEM	Infectivity to mice
I. c507 vs. *Δc43* sporozoite numbers in *LacZ* and *LRIM1* kd mosquitoes						
c507	*LacZ*	7,790	785	8,622	673	6 of 6
c507	*LRIM1*	17,343	1,988	16,060	665	6 of 6
Δc43	*LacZ*	0	0	0	0	0 of 10
Δc43	*LRIM1*	580	64	65	13	0 of 11
II. c507 vs. Pb^{Pfc43} sporozoite numbers in *LacZ* and *LRIM1* kd mosquitoes						
c507	*LacZ*	13,836	198	ND	ND	2 of 2
c507	*LRIM1*	23,504	491	ND	ND	1 of 1
Pb^{Pfc43}	*LacZ*	346.86	9	ND	ND	0 of 1
Pb^{Pfc43}	*LRIM1*	456.41	13	ND	ND	0 of 1

I. Mean oocyst and salivary gland sporozoite numbers in *LacZ* and *LRIM1* knockdown *A. coluzzii* mosquitoes infected with *c507 or Δc43* parasites, obtained from 2 to 4 biological replicates. In each replicate, the mean number of sporozoites was calculated from three pools of ten homogenised midguts or salivary glands at 15 and 21 days post blood feeding respectively. Parasite infectivity to mice was assessed upon blood feeding of at least 30 mosquitoes on C57BL/6 mice at 21-22 days post blood feeding. Parasitaemia was monitored until 14 days post mosquito bite. SEM represents the standard error of the mean.

II. The mean number of midgut sporozoites in *LacZ* and *LRIM1* knockdown *A. coluzzii* mosquitoes infected with Pb^{Pfc43} or *c507* parasites was calculated from two replicates obtained from suspensions of 10-12 homogenised midguts at day 15 post blood feeding. Parasitaemia was monitored until 14 days post mosquito bite.

Table S6. Sporozoite development and infectivity upon ookinete injection in the haemocoel

	Salivary gland sporozoites		
Parasite	Mean	SEM	Infectivity to mice
c507	6530 (2,934/10,117/6540)	1693	6/6
Δc43	0 (0/0/0)	0	0/6

Mean salivary gland sporozoites at 21 days post *A. coluzzii* haemocoel inoculation with *c507 wt* or Δ*c43* ookinetes, obtained from 3 biological replicates. Data from independent biological replicates are presented in brackets. Infectivity of sporozoites was assessed by infected mosquito bite back experiments of C57/BL6 mice at day 21 post haemocoel inoculation. Parasitaemia was monitored for 14 days post mosquito bite. SEM represents the standard error of the mean.

Table S7. Enriched Gene Ontologies in differentially regulated genes at 24 hpbf between *Δc43* and *c507* parasite lines

Description	Fold enrichment	P value	Bonferroni correction
Biological process:			
Locomotion	5.92	4.27E-06	0.001
Movement in host environment / symbiotic organism	6.39	6.94E-06	0.002
Entry into host or symbiotic organism	7.1	1.03E-05	0.002
Interaction with host	5.7	1.83E-05	0.004
Multi-organism process	4.93	2.31E-05	0.005
Interspecies interaction between organisms / mutualistic symbionts through parasitism	5.41	2.83E-05	0.006
Entry into host cell / symbiotic cell of another organism	7.1	3.84E-05	0.009
Cellular component:			
Microneme	9.56	4.61E-06	0.004
Apical part of cell	4.88	9.56E-06	0.008
Apical complex	4.99	5.54E-05	0.004

Table S8. *P. falciparum* transmission blocking effect of the α-Pfc43^{opt} antibody in SMFA

Assay	α-Pfc43^{opt} concentration (µg/mL)	Number of midguts	Prevalence (%)	*P*-value prevalence	Infection Intensity			
					Arithmetic mean (m)	Median (M)	Oocyst range	*P*-value intensity
Total	0	137	70.8		22.05	5.0	0-332	
	50	119	63.9	0.2843	13.73	4.0	0-133	0.2926
	125	171	44.4	<0.0001	9.45	0.0	0-250	<0.0001
	250	171	45.6	<0.0001	5.25	0.0	0-92	<0.0001
Replicate 1	0	28	89.3		71.29	47.0	0-332	
	125	28	46.5	0.0013	34.82	0.0	0-230	0.0019
	250	26	46.2	0.0010	8.731	0.0	0-92	<0.0001
Replicate 2	0	25	68		4.56	2.0	0-18	
	125	40	67.5	0.1292	2.02	1.0	0-52	0.0586
	250	40	45.0	0.0468	1.67	0.0	0-45	0.0180
Replicate 3	0	40	55.0		7.27	2.0	0-53	
	50	76	57.9	0.8444	11.64	2.0	0-133	0.4494
	125	49	44.9	0.3974	10.00	0.0	0-250	0.2105
	250	58	36.2	0.0971	4.57	0.0	0-53	0.0725
Replicate 4	0	44	75.0		16.04	8 .0	0-58	
	50	43	74.4	1.0000	19.52	11.5	0-83	0.5907
	125	54	37.0	0.0002	3.1	0.0	0-21	<0.0001
	250	47	57.4	0.1207	9.38	2.0	0-42	0.0185

Oocyst data at 7 days post blood feeding from *P. falciparum* SMFAs using the α-Pfc43^{opt} antibodies. P values for infection prevalence were calculated using the Fisher's exact test and P values for infection intensities on the basis of the median number of oocysts was calculated using the Mann-Whitney U test.

Table S9. *P. berghei* transmission blocking efficacy of the α-Pbc43 antibody in SMFA

Assay	Antibody/concentration (µg/mL)	Number of midguts	Prevalence (%)	*P*-value[1] (Prevalence)	Infection Intensity<br>Arithmetic mean (m)	Median (M)	Oocyst range	*P*-value[2] (Intensity)
Total	α-Pbc43/50	110	58.2		13.0	2.0	0-98	
	UPC10/50	126	76.2	0.0035	21.9	9.0	0-172	0.001
	α-Pbc43/100	113	52.2		6.25	1.0	0-78	
	UPC10/100	126	81.0	<0.0001	22.9	14.5	0-131	<0.0001
	α-Pbc43/250	119	27.7		2.3	0.0	0-27	
	UPC10/250	128	80.5	<0.0001	23.7	14.0	0-189	<0.0001
Replicate 1	α-Pbc43/50	37	64.9		24.3	21.0	0-98	
	UPC10/50	26	76.9	0.4059	46.3	40.0	0-172	0.0429
	α-Pbc43/100	42	50.0		8.6	0.5	0-78	
	UPC10/100	35	94.3	<0.0001	43.3	42.0	0-131	<0.0001
	α-Pbc43/250	32	9.4		0.50	0.0	0-8	
	UPC10/250	45	82.2	<0.0001	34.4	23.0	0-189	<0.0001
Replicate 2	α-Pbc43/50	35	57.1		5.1	1.0	0-48	
	UPC10/50	50	68.0	0.3630	19.0	10.5	0-136	0.0068
	α-Pbc43/100	50	56.0		5.1	1.5	0-64	
	UPC10/100	50	74.0	0.0928	17.2	12.0	0-86	0.0004
	α-Pbc43/250	50	38.0		3.5	0.0	0-22	
	UPC10/250	43	79.1	<0.0001	20.8	9.0	0-162	<0.0001
Replicate 3	α-Pbc43/50	38	52.6		6.5	2.0	0-40	
	UPC10/50	50	84.0	0.0020	12.0	6.0	0-79	0.0052
	α-Pbc43/100	21	47.6		4.3	0.0	0-23	
	UPC10/100	41	79.0	0.0222	12.5	8.0	0-44	0.0067
	α-Pbc43/250	37	29.7		2.2	0.0	0-27	
	UPC10/250	40	80.0	<0.0001	14.6	7.0	0-70	<0.0001

Oocyst data at 10 dpbf from *P. berghei* SMFA's using the α-Pbc43 antibodies. *P. berghei* SMFA's with UPC10 antibodies served as a control. P values for infection prevalence were calculated using the Fisher's exact test and P values for infection intensities on the basis of the median number of oocysts was calculated using the Mann-Whitney U test.

Table S10. Primers for RT-PCR, qRT-PCR, protein expression and generation of transgenic parasites

Primer name	Sequence (5′ to 3′)	Description
Pfc43 RT-PCR F	GTTGATATAAAACAAGATGATTTTACTAATGG	
Pfc43 RT-PCR R	GAAATATTTTATACTAGAATATATGGAAGAACCAAACAT	
Pfs25 RT-PCR F	GCGAAAGTTACCGTGGATACTG	
Pfs25 RT-PCR R	ACTCCAGTTTTAACAGGATTGCT	
PfCSP RT-PCR F	TGGACAAGGTCACAATATGCCA	
PfCSP RT-PCR R	ACGACATTAAACACACTGGAACA	
Pbc43 RT-PCR F	ATACGGGAATCCATCAACCA	
Pbc43 RT-PCR R	ACTTCAAACGACCCTTGTGC	
Pbs28 RT-PCR F	AATGCACAGGTACAGGAGAAACTAAAT	
Pbs28 RT-PCR R	CACACTCATAATGTTTTCCAGTCAATT	
PbCTRP RT-PCR F	AGAGAAGAAGATTGCCCAACAG	
PbCTRP RT-PCR R	ATCGGATCATTTGCATCGATAAC	
GFP RT-PCR F	CCTGTCCTTTTACCAGACAACCA	
GFP RT-PCR R	GGTCTCTCTTTTCGTTGGGATCT	
Pbc43 qRT-PCR F	TTGAATTAGCACAAGGGTCGTTTGAA	
Pbc43 qRT-PCR R	CTTCTTTAGCTGGGGTATTATCGTGC	
GFP qRT-PCR F	CCTGTCCTTTTACCAGACAACCA	
GFP qRT-PCR R	GGTCTCTCTTTTCGTTGGGATCT	
S7 qRT-PCR F	GTGCGCGAGTTGGAGAAGA	
S7 qRT-PCR R	ATCGGTTTGGGCAGAATGC	
LRIM1 qRT-PCR F	AAGTTGACCGGCCAGAATGAGGAAG	
LRIM1 qRT-PCR R	GGCAGATCCTCGCAGCAGTACG	
APL1C qRT-PCR F	GCTTCACTTTTTGGCGCT	
APL1C qRT-PCR R	CAGGCTGAGTTGAGACAGGA	
TEP1 qRT-PCR F	AAAGCTAGCAATTTGTTGCGTCA	
TEP1 qRT-PCR R	TTCTCCCACACACCAAACGAA	
Pfc43^{opt} IF F	GACAAGCTTGCGGCCGCAAAGGACGACGGCCAAAGAC	*E. coli* expression
Pfc43^{opt} IF R	TGCTCGAGTGCGGCCGCCAGGATGGTGGTGTAGAGCTTGCA	*E. coli* expression
Pbc43^{opt} IF F	GACAAGCTTGCGGCCGCAATCAACGGTTCCGGCAACAC	*E. coli* expression
Pbc43^{opt} IF R	TGCTCGAGTGCGGCCGCCAGCAGGGTGTAGTTCTTCTTGCA	*E. coli* expression
Pbc43 LIC F	GACGACGACAAGATGATAAATGGTTCTGGTAATACAG	SF9 expression
Pbc43 LIC R	GAGGAGAAGCCCGGTTTGCTATTAGACATTAGCAATGTG	SF9 expression
P1 F	TTGGGCCCGAAATTGTGAGTCTTGTATTATTTCAATAG	Disruption upstream target ApaI
P2 R	CCAAGCTTGCAAAATAACAATAACAAATTCCTTTAAG	Disruption upstream target HindIII
P3 F	TGAATTCAGCTGGGATTGAATTAGCACAAGGGTCG	Disruption downstream target EcoRI
P4 R	TTGGATCCGACATTAATTTAGAGGCTATGCTATTAG	Disruption downstream target BamHI
INT F1 (P5)	GATCGAAATAAATAATTTTGAATAAG	Diagnostic primer WT and KO/*c507*

WT R (P6)	GTACTACTTCACCTGTATTACCAG	Diagnostic primer WT/*c507*
TgDHFR 5′UTR R (P7)	GATGTGTTATGTGATTAATTCATACAC	Diagnostic primer KO/*c507*
PlasmoGEM QCR1 F(P8)	AGGAATTTGTTATTGTTATTTTGC	Diagnostic primer WT/*1804cl1*
PlasmoGEM QCR2 R(P9)	CTGTCTACACTTATTGTGCC	Diagnostic primer WT/*1804cl1*
PlasmoGEM GW1 F(P10)	CGGGGCCCTTATGCATAATC	Diagnostic primer KO/*1804cl1*
PlasmoGEM GT R (P11)	GTGCACGTGCGTTAGTGGG	Diagnostic primer KO/*1804cl1*
P12 F	CCAAGCTTGGGTAATGAATATACATGGATATATG	*Pfc43* complement upstream target HindIII
P13 R	GGGGGCCCTATCGTTTTTTTACTTGAAAAATAAAA	*Pfc43* complement upstream target ApaI
P14 F	TTGGGCCCATGCAGAAACGAATTTATGTATCATTA	*Pfc43* CDS ApaI
P15 R	GGCCGCGGTTACAATATGTATATAACAAATAACATTAAT	*Pfc43* CDS SacII
P16 F	GGCCGCGGTAATTATATGTAAAGCAAAGCATTTAGTGATTAT	*Pbc43 3'UTR* for *Pfc43* complement SacII
P17 R	GGCCGCGGTAAATACACCCTATATTTCATATTATTGGAAT	*Pbc43 3'UTR* for *Pfc43* complement SacII
P18 F	TTCTCGAGAGCTGGGATTGAATTAGCACAAGGGTCGTTTG	*Pfc43* complement downstream target XhoI
P19 R	TTCCCGGGGGTCTACAATTTTTTATGTAGATGAAA	*Pfc43* complement downstream target SmaI
INT F2 (P20)	GGATCCTTTGGATTGGTCTTTTCCCATAAAG	Diagnostic primer WT, *Pfc43* and *Pbc43* comp
Pfc43 INT R (P21)	TCCTCTCCGTGACGTTGGCCATCATCTTTG	Diagnostic primer *Pfc43* complement
P22 F	TTAAGCTTGAGAGCCCTCTTTAGGTGTTTAAG	*Pbc43* WT complement upstream target HindIII
P23 R	GGCCGCGGTAAATACACCCTATATTTCATATTA	*Pbc43* WT complement upstream target SacII
P24 F	TTCTCGAGTAATTATATGTAAAGCAAAGCATTT	*Pbc43* WT complement downstream target XhoI
P25 R	TTCCCGGGTAAATACACCCTATATTTCATATTATT	*Pbc43* WT complement downstream target SmaI
Pbc43 INT R (P26)	CACATATATAATACAGTATGCTACTTTTAC	Diagnostic primer *Pbc43* WT complement

Where appropriate, target restriction sites are shown as underlined italics and restriction site overhangs are also shown. The appropriate restriction enzyme is presented in the description column. F, forward; R, reverse; LIC, ligation independent cloning; IF, in-fusion; INT, integration; WT, wild-type; KO, knockout; UTR, untranslated region. All primers are listed in a 5′ to 3′ direction.

Supplementary materials and methods

Sequence analysis

Plasmodium DNA and protein sequences were retrieved from PlasmoDB (http://plasmodb.org/plasmo/) and aligned using ClustalW2 in the BioEdit sequence alignment editor program. Signal peptide and transmembrane domains were predicted using SignalP4.0 (1) and TMHMM Server v. 2.0 (2), respectively.

P. berghei strains and culturing

P. berghei ANKA lines used include: *cl15cy1* (*2.34*), the reference parent line of *P. berghei* ANKA, *507m6cl1* (*c507*) constitutively expressing GFP integrated into the *230p* gene locus (*PBANKA_0306000*) without a drug selectable marker (3), *1804cl1* (*c1804*) constitutively expressing mCHERRY integrated into the *230p* locus without a drug selectable marker (4), and *2.33* (5), a non-gametocyte producer line. All lines were maintained in 8-10 week old CD1 and/or Balb/c female mice by serial passaging. Culturing and purification of *P. berghei* ABS, gametocytes and ookinetes were carried out as described previously (3).

P. falciparum culturing

P. falciparum NF54 was cultured as described previously (6). Briefly, ABS and gametocytes were cultured using hRBCs of various blood groups in the following order of preference: O+ male, O+ female, A+ male and A+ female. Set up of *Pf*NF54 asexual culture was performed in T25 cm^2 flasks containing a final volume of 500 µL of hRBCs (0.3-4% infection) and 10 mL of CM RPMI-1640-R5886 (Sigma), 0.05 g/L Hypoxanthine, 0.3 mg/L L-glutamine powder (G8540-25G Sigma) and 10% (v/v) sterile human serum of A+ serotype), kept at 37°C incubation and supplemented with "malaria gas" [3% O_2/5% CO_2/92% N_2 (BOC Special Gases, cat. no. 226957-L-C)]. Gametocyte cultures were initiated by diluting the continuous asexual culture (3-4% ring forms) to 1% ring forms by the supply of fresh hRBCs. Gametocyte cultures were kept at constant temperature of 37°C until day 14 ensuring daily exchange of around 75% of the medium per flask.

RT-PCR and quantitative RT-PCR

Total RNA was extracted from *P. falciparum* and *P. berghei* parasites using Trizol reagent (ThermoFisher) according to the manufacturer's instructions. cDNA was synthesized using the Primescript Reverse Transcription Kit (Takara) after Turbo DNase (ThermoFisher) treatment. For RT-PCR, the resulting cDNA was used in the PCR of *P. falciparum* and *P. berghei PIMMS43* using gene specific primers (**Table S10**). *P. falciparum Pfs25* and *PfCSP*, and *P. berghei P28* and *CTRP* served as stage specific controls. Constitutively expressed *GFP* in *P. berghei* was used as an internal control. For qRT-PCR, SYBR green (Takara) and gene specific qRT-PCR primers (**Table S10**) were used according to the manufacturer's guidelines. Expression of *PbPIMMS43* was normalized against *GFP* and expression of *TEP1*, *LRIM1* and *APL1C* was normalized against *S7* using the $\Delta\Delta$Ct method.

Expression and purification of recombinant PIMMS43 in *E. coli*

PfPIMMS43 and *PbPIMMS43* was codon optimized for expression in *E. coli* (GeneArt, ThermoFisher) and termed $Pfc43^{opt}$ and $Pbc43^{opt}$, respectively. $Pfc43^{opt}$ (aa 25-481) and $Pbc43^{opt}$ (aa 22-327) fragments that both exclude the signal peptide and the C-terminal hydrophobic domain were amplified with primers (**Table S10**) and cloned into a NotI digested protein expression vector plasmid, pET-32b (which carries N and C-terminal 6xHistidine tags) (Novagen) by In-Fusion Cloning (Takara). Shuffle T7 *E. coli* cells (NEB) containing the recombinant protein expression plasmid were grown at 30°C and induced with 1 mM isopropyl-1-thio-β-d-galactopyranoside at 19°C for 16 h. Cells were harvested and lysed using bugbuster-lysonase (Novagen) containing protease inhibitors (cOmplete EDTA-free, Roche). Cell debris were removed by centrifugation. Both proteins were expressed as His-fusion proteins and hence purified by cobalt affinity chromatography using TALON® metal affinity resin (Takara). The $Pfc43^{opt}$ recombinant protein was soluble and purified under native conditions in phosphate buffered saline (PBS), pH 7.4. The $Pbc43^{opt}$ recombinant protein was extracted from inclusion bodies using the Inclusion Body Solubilization Reagent (ThermoFisher). The solubilized protein was then purified under denaturing conditions in 8M urea in PBS, pH 7.4. Refolding of $Pbc43^{opt}$ was carried out in decreasing concentrations of urea in PBS. Protein samples were analyzed by SDS-PAGE to determine purity prior to their use for immunization in rabbits for the generation of the affinity purified polyclonal antibodies α-$Pfc43^{opt}$ and α-$Pbc43^{opt}$ (Eurogentec).

Expression and purification of recombinant PIMMS43 in Sf9 cells

PbPIMMS43 (aa 22-331) that excludes the signal peptide and includes four amino acids of the C-terminal hydrophobic domain was amplified from 24 h *in vitro* ookinetes cDNA using primers in **Table S10**. This fragment was cloned by ligation independent cloning into the linearized pIEX-10 EK-LIC vector which carries a C-terminal 10xHis tag (Novagen) to generate pIEX-10: *Pbc43*-SP/TM. A stable line expressing the

recombinant protein was generated by co-transfection of pIEX-10: *Pbc43*-SP/TM and pIEX-10:Neo plasmid(7) using the Cellfectin® II Reagent (ThermoFisher) according to the manufacturers' guidelines. pIEX-10:Neo plasmid carries the neomycin resistance cassette and provides resistance to the antibiotic G418 (Sigma). The recombinant protein was extracted from cells using lysis buffer (1XPBS, 1% v/v Triton X-100, pH 7.4) containing benzonase (Novagen) and protease inhibitors. The His-tagged recombinant PbPIMMS43 protein was insoluble and extracted by solubilization in 8M urea in PBS, pH 7.4. The protein was purified using TALON® metal affinity resin under denaturing conditions in 8M urea in PBS, pH 7.4. Bound proteins were eluted using denaturing elution buffer. Refolding of Pbc43 was carried out in decreasing concentrations of urea in PBS. Protein samples were analyzed by SDS-PAGE to determine purity prior to their use for immunization in rabbits in the generation of the affinity purified polyclonal antibody α-Pbc43^{Sf9} (Eurogentec).

Western blot analysis
Whole cell lysates were prepared by suspending purified parasite pellets in whole cell lysis buffer (1XPBS, 1% v/v Triton X-100) containing protease inhibitors. Using the previously described method (28), 24 h *in vitro* ookinetes were subjected to cellular fractionation to obtain the soluble, Triton-Soluble and insoluble cell fractions. Protein samples were then boiled under non-reducing or reducing (3-5% v/v 2-mercapthoethanol) conditions in Laemilli buffer and separated using 10% sodium dodecyl sulfate polyacrylamide gel electrophoresis. Separated proteins were then transferred to a PVDF membrane (GE Healthcare). Proteins were detected using α-Pbc43^{opt} (1:100), α-Pfc43^{opt} (1:100), goat α-GFP (Rockland chemicals) (1:100) and 13.1 mouse monoclonal α-P28 (8) (1:1000) antibodies. Secondary horseradish peroxidase (HRP) conjugated goat α-rabbit IgG, goat α-mouse IgG antibodies (Promega) and donkey α-goat IgG (Abcam) were used at 1: 10,000, 1: 10,000 and 1: 5,000 dilutions, respectively. All primary and secondary antibodies were diluted in 3% milk-PBS-Tween (0.05% v/v) blocking buffer.

Indirect immunofluorescence assay
IFA's were carried out on blood bolus parasites, ookinetes invading the midgut epithelium, oocysts, oocyst sporozoites, and salivary gland sporozoites. For IFA's on blood bolus parasites, midguts of blood fed mosquitoes at 1 hpbf were dissected, and the blood boluses were collected. Blood bolus was washed in PBS prior to fixation in 4% paraformaldehyde (PFA) in PBS for 30 min. Fixed parasites were smeared on glass slides, allowed to air dry, permeabilized with 0.2% v/v Triton X-100, and blocked in a 3% w/v bovine serum albumin (all diluted in PBS). For IFA's on ookinetes invading the midgut epithelium at 24-30 hpbf, or young oocysts at 2 dpbf, midguts of blood fed mosquitoes were dissected, and blood boluses were discarded. The midgut epithelium was fixed in 4% PFA in PBS for 45 min and washed thrice in PBS for 10 min each. Midgut epithelium was permeabilized and blocked for 1 h with 1% w/v BSA, 0.1% v/v Triton X-100 in PBS. For IFA's on sporozoites, infected midguts and salivary glands were dissected, and tissues were homogenized to release sporozoites. Sporozoites were fixed, blocked and permeabilized as that used for blood bolus parasites. Samples were then stained in blocking solution with primary antibodies (α-Pfc43^{opt}, 1:300; 4B7 mouse monoclonal α-Pfs25 (9), 1:1000; 2A10 mouse monoclonal α-PfCSP (10), 1:200; α-Pbc43^{opt}, 1:100; 13.1 mouse monoclonal α-P28, 1:1000; and 3D11 mouse monoclonal α-PbCSP (11), 1:100. Alexa Fluor (488 and 568) conjugated secondary goat antibodies specific to rabbit or mouse (ThermoFisher) were used at a dilution of 1:1000. 4′,6-diamidino-2-phenylindole (DAPI) was used to stain nuclear DNA. Images were acquired using a Leica SP5 MP confocal laser-scanning microscope. Images underwent processing by deconvolution using Huygens software and were visualized using Image J.

Generation of transgenic parasites
Partial ko of *P. berghei* c*43* CDS was carried out by double crossover homologous recombination in the *c507* and *1804cl1* lines. For partial disruption in the *c507* line, a 765 bp ApaI/HindIII 5' homology arm and a 528 bp EcorI/BamHI 3' homology arm was amplified from *P. berghei 2.34* genomic DNA using the primer pairs P1/P2 and P3/P4 respectively. These fragments were cloned into the pBS-TgDHFR vector which carries a modified *Toxoplasma gondii* dihydrofolate gene (*TgDHFR/TS)* cassette that confers resistance to pyrimethamine(12). The targeting cassette was released by ApaI/BamHI digestion and it allows ko of 50% of *P. berghei* c*43* CDS at the 5' region. For partial disruption in the *1804cl1* line, the target vector containing the human *DHFR* selection cassette was used (kindly provided by plasmoGEM, vector design ID, PbGEM-042760; http://plasmogem.sanger.ac.uk/). *hDHFR* confers resistance to the drugs pyrimethamine and WR92210. The targeting cassette was released by NotI digestion and allows ko of 74% of *PbPIMMS43* CDS leaving a small part of the 3' region of the CDS.
To express *P. falciparum c43* in *P. berghei*, the transgenic parasite *Pb*^{Pfc43} was created in the *c507* line. The *Pfc43* replacement construct was generated using the plasmid pL0035 which carries the *hDHFR* selection cassette (13). A 1.7 kb HindIII/ApaI fragment upstream of the *PbPIMMS43* coding DNA sequence was amplified using primer pair P12/P13 and cloned upstream of the pL0035 selectable marker cassette. The 1.5

kb *Pfc43* CDS was amplified from cDNA using the ApaI and SacII primer pair P14/P15 and cloned downstream of the 1.7kb fragment. A 518 bp region corresponding to the *3'UTR*, downstream of the *PbPIMMS43* stop codon, was amplified as a SacII fragment using primers P16/P17 and cloned downstream of the 1.5 kb *Pfc43* CDS. Downstream of the pL0035 selectable marker, a 700 bp region corresponding to part of the *PbPIMMS43* coding region and part of the *3'UTR* was amplified using primers P18/P19 and cloned as a XhoI and SmaI fragment. This replacement vector was linearized by HindIII and SmaI digestion.
To re-introduce *PbPIMMS43* into the *Δc43* ko parasite, the transgenic parasite $\Delta c43::c43^{wt}$ was created. A 3.5 kb upstream region that includes the *PbPIMMS43* ORF and its *5'UTR* and *3'UTR* was amplified as a HindIII and SacII fragment using primers P22/P23. A 518 bp downstream region corresponding to the *PbPIMMS43 3'UTR* was amplified as a XhoI and SmaI fragment using primers P24/P25. These fragments were cloned into the pL0035 vector and served as homology regions for homologous recombination at the *Δc43* ko locus in the *c507* line. This replacement vector was linearized by HindIII and SmaI digestion.
Transfection of linearized constructs, selection of transgenic parasites and clonal selection was carried out as described previously (3).

Genotypic analysis of transgenic parasites
Purified blood stage parasites were obtained after white blood cells removal using hand packed cellulose (Sigma) columns and red blood cell lysis in 0.17M NH_4Cl on ice for 20 min. Genomic DNA was extracted from parasites using the DNeasy kit (Qiagen). Detection of successful integration events or maintenance of the unmodified locus was performed by PCR on genomic DNA using primers listed in **Table S10**. Blood stage parasites within agarose plugs were lysed in lysis buffer (1XTNE, 0.1 M EDTA pH 8.0, 2% (v/v) Sarkosyl, 400μg/mL proteinase K) to release nuclear chromosomes. Southern blot analysis on pulsed field gel electrophoresis separated chromosomes (Run settings: 98 volts, 1-5 mins pulse time for 60 h at 14 °C) was carried out with a probe targeting the *TgDHFR/TS-P. berghei DHFR 3'UTR*, obtained by HindIII and EcoRV digestion of the pBS-TgDHFR plasmid.

Phenotypic assays
Exflagellation assays were performed as described previously (14). Briefly blood from a high gametocytemia mouse was added in a 1:40 ratio to ookinete medium (RPMI 1640, 20% v/v FBS, 100 μM xanthurenic acid, pH 7.4), and exflagellation was counted in a standard haemocytometer under a light microscope.*In vitro* conversion assays were performed as previously described (14). For *in vivo* conversion assays, the blood bolus of 10 mosquitoes at 17-18 hpbf was pelleted, washed in PBS and resuspended in 50 μL of fresh ookinete medium. The suspension was then incubated with a Cy3-labelled 13.1 mouse monoclonal α-P28 (1:50 dilution) for 20 min on ice. The conversion rate was calculated as the percentage of Cy3 positive ookinetes to Cy3 positive macrogametes and ookinetes.
Ookinete motility assays were performed as described (15). Briefly, 24 h *in vitro* ookinete culture was added to Matrigel (BD biosciences), dropped onto a slide, sealed with nail varnish, and allowed to set at RT for 30 min. Time-lapse microscopy (1 frame every 5 seconds, for 10 min) of ookinetes were taken on a Leica DMR fluorescence microscope and a Zeiss Axiocam HRc camera controlled by the Axiovision (Zeiss) software. The speed of individual ookinetes was measured using the manual tracking plugin in the Icy software package (http://icy.bioimageanalysis.org/).

Ookinete injections in mosquito haemocoel
24 h *in vitro* ookinetes was adjusted with RPMI 1640 to achieve an injection concentration of 800 ookinetes per mosquito as described previously (16). This was injected into the thorax of *A. coluzzii* mosquitoes using glass capillary needles and the Nanoject II microinjector. Salivary gland sporozoites were counted at 21 dpbf as described below.

Gene silencing
cDNA was prepared from total RNA extracted (as described above) from *A. coluzzii* midgut infected with *P. berghei c507*, at 24 hpbf. The cDNA was used in the amplification of *CTL4*, *LRIMI* and *TEPI* using primers with T7 overhangs as reported in (17, 18). The resulting T7 PCR products and the T7 high yield transcription kit (ThermoFisher) was used to produce dsRNA. DsRNA was purified using the RNeasy kit (Qiagen) and 0.2 μg in 69 nL was injected into the thorax of *A. coluzzii* mosquitoes using glass capillary needles and the Nanoject II microinjector (Drummond Scientific). Injected mosquitoes were left for 2-3 days before *P. berghei* infection.

RNA-sequencing library preparation
Three replicate infections of *A. coluzzii* mosquitoes with the *Δc43* and *c507 P. berghei* lines were performed and infected midguts were dissected at 1 and 24 hpbf. Total RNA was extracted as described elsewhere and was used for RNA sequencing by Genewiz (New Jersey, US) using the NEB Ultra prep kit and an Illumina

HiSeq platform with 150x2 paired-end reads. Prior to the RNA sequencing, successful infection of the midgut epithelium was confirmed by P28-staining of parasites in 5 midguts from each replicate infection: Replicate 1, *c507* median 536 (458, 635, 495, 536, 598), *Δc43* median 501 (419, 436, 501, 605, 520), Replicate 2, *c507* median 386 (386, 421, 350, 258, 408), *Δc43* median 389 (347, 411, 389, 369, 402) and Replicate 3, *c507* median 548 (501, 426, 548, 603, 551), *Δc43* median 495 (495, 504, 521, 465, 436).

NGS RNA-sequencing-Data processing and analysis

RNA-Seq reads were mapped using HiSat2 v2.0.5 (19) with default parameters to the *A. gambiae* genome (AgamP4 assembly) (20) and the *P. berghei ANKA* (21). Transcript abundance was quantified as fragments per kilobase per million reads (FPKM) using Cufflinks v2.2.1 (22) on the *A. gambiae* (Anopheles-gambiae-PEST_BASEFEATURES_AgamP4.9.gtf) and *P. berghei* (PlasmoDB-39_PbergheiANKA.gff) annotation sets. Differential expression analysis was performed using Cuffdiff v.2.2.1 (23). The sequencing data were uploaded to the Galaxy web platform (an open source, web-based platform for data intensive biomedical research), and we used the VectorBase Galaxy server (https://galaxy.vectorbase.org) to analyze the data (24). Data are derived from three independent biological replicates, each of which included three technical replicates. To filter out the biological or technical noise from the actively expressed genes, an FPKM cutoff was selected that is based on an implementation of the zFPKM normalization method described previously (25). Functional classification of *P. berghei* differential regulated genes were performed in PlasmoDB (http://plasmodb.org/plasmo/) using the *P. berghei* full genome as a reference genome. PANTHER (v13.1; http://pantherdb.org) (26) was used for functional classification of *A. gambiae* differentially regulated genes. The RNA sequencing data were deposited to and can be downloaded from the European Nucleotide Archive with experiment codes ERX3197375-410.

Population genetics analysis

The genome sequences of 1,509 African *P. falciparum* samples determined in the context of the *P. falciparum* Community Project were obtained from the MalariaGen website (http://www.malariagen.net/data). They include samples from 11 African countries including Gambia (73), Guinea (124), Mali (87), Burkina Faso (56), Ghana (478), DR of the Congo (279), Uganda (12), Kenya (52), Tanzania (68), Malawi (262) and Madagascar (18). Call of SNPs found in *PfPIMMS43* and *Pfs47* exonic sequences were based on the 3D7 reference genome assembly version 6.0 (Jan. 2016). F_{ST} values were calculated using the R (v.3.2.1) packages gdsfmt and SNPRelate (27) by considering (a) all SNPs across each gene and all populations within a given country and (b) each individual SNP sampled from populations in each of the 11 African countries (F_{ST} total) and in pairwise country comparisons.

***P. berghei* mosquito infections**

Mosquitos were infected with *P. berghei* by utilizing direct feeding assays (DFAs) or standard membrane feeding assays (SMFAs). DFAs were carried out by mosquito feeding on mice with 5-6% parasitaemia and 1-2% gametocytaemia. SMFAs were carried out as described previously (28). Briefly, for each feed, 350 µL of heparanized *P. berghei* ANKA *2.34* infected blood from mice with 5-6% parasitaemia and 2-3% gametocytaemia was mixed with 150 µL of PBS containing either α-Pbc43^{Sf9} or the isotopic monoclonal UPC10 (negative control; Sigma) antibodies to yield final antibody concentrations of 50, 100 and 250 µg/mL. Infected mosquitoes were maintained at 19-21°C, 70-80% humidity, 5% sucrose and 12/12 hours light/dark cycle.

Midguts tissues were dissected at 4-10 dpbf and fixed in 4% PFA in PBS. Fixed midguts were mounted in Vectashield® (VectorLabs) and oocysts or melanised ookinetes were enumerated using light and/or fluorescence microscopy. Oocyst images and sizes were also analyzed using fluorescence microscopy. Oocyst and salivary gland sporozoite numbers at 15 and 21 dpbf respectively were counted using a standard haemocytometer, in 3 technical replicates of homogenates of 10 *P. berghei* infected *A. coluzzii* midguts or salivary glands.

Finally, in mosquito to mouse transmission assays, at least 30 *P. berghei* infected mosquitoes were allowed to feed on 2-3 anaesthetized C57/BL6 mice at 20-22 dpbf. Parasitaemia was monitored up until 14 days post mosquito bite by Giemsa stained tail blood smears.

***P. falciparum* mosquito infections**

Mosquitos were infected with *P. falciparum* by SMFAs as described previously (6). Briefly, gametocytemia and density of viable mature stage V gametocytes at day 14 post-induction were assessed by Giemsa stained blood smears and by testing *in vitro* exflagellation of male gametocytes respectively. Day 14 stage V gametocytes cultures were pooled in a pre-warmed tube containing 20% v/v uninfected serum-free hRBCs and 50% v/v heat-inactivated human serum to a final volume of 300 µL. The mixture was immediately transferred to pre-warmed glass feeders kept a constant temperature of 37°C and mosquitoes were allowed to feed. For transmission blocking assays, α-Pfc43^{opt} antibodies were added to the gametocytemic blood mix

to final antibody concentrations of 50, 125 and 250 µg/mL, in a final volume of 300 µL. Blood fed mosquitoes were maintained at 27°C, 70% humidity, 5% sucrose and 12/12 hours light/dark cycle. Infected midgut tissues at 10 dpbf were dissected and analyzed as above.

Statistical analysis

Statistical analyses for exflagellation, ookinete conversion and motility assays were performed using a two-tailed, unpaired Student's *t*-test. For statistical analyses of the oocyst or melanized parasite load (infection intensity) and presence of oocysts (infection prevalence), *p* values were calculated using the Mann-Whitney test and the Fisher's exact test, respectively. Statistical analyses were performed using GraphPad Prism v7.0 and v8.0. The generalized linear mixed model (GLMM) was used to also determine statistical significance in oocyst infection intensity in transmission blocking assays. GLMM analyses were performed in R (version 2.15.3) using the Wald Z-test on a zero-inflated negative binomial regression (glmmADMB). The various treatments were considered as covariates and the replicates as a random component. Fixed effect estimates are the regression coefficients.

Supplementary Dataset legends

Dataset S1 (separate file). RNAseq transcription profiling of *P. berghei Δc43* and *c507* lines in the *A. coluzzii* midgut

Transcription profiles are assessed at (**A**) 1 hpbf and (**B**) 24 hpbf. FPKM is transcript abundance quantified as fragments per kilobase per million reads in each of the Δc43 and c507 parasite lines. The log_2-transformed ratio of *Δc43* to *c507* FPKM data for each gene is shown (log2 (Δc43/c507)). P values (P value), adjusted P values (q value) and statistical significance (q<0.005; yes or no) are indicated. Data are derived from three independent biological replicates, each of which included three technical replicates.

Dataset S2 (separate file). RNAseq transcription profiling of *A. coluzzii* midgut responses to *P. berghei Δc43* and *c507* infections

Transcription profiles are assessed at (**A**) 1 hpbf and (**B**) 24 hpbf. FPKM is transcript abundance quantified as fragments per kilobase per million reads in each of the *A. coluzzii* genes infected with *Δc43* (Ac Δc43) and *c507* (Ac c507) parasite lines. The log_2-transformed ratio of FPKM values for each *A. coluzzii* gene in infections with *Δc43* to infections with *c507* is shown (log2 (Ac Δc43/Ac c507)). P values (P value), adjusted P values (q value) and statistical significance (q<0.005; yes or no) are indicated. Data are derived from three independent biological replicates, each of which included three technical replicates.

Dataset S3 (separate file). Fixation index analysis of *P. falciparum PIMMS43* and *Pfs47*

Fixation index (F_{ST}) values for all SNPs in (**A**) *PfPIMMS43* and (**B**) *Pfs47* genes are calculated from 1,509 parasites samples from 11 African countries. F_{ST} values from the analysis of all samples together (F_{ST} total) and from country pairwise comparisons are shown. NA indicates monomorphic sites. SNP IDs and their position, reference and alternative alleles, and amino acid changes are indicated.

Supplementary References

1. Petersen TN, Brunak S, von Heijne G, & Nielsen H (2011) SignalP 4.0: discriminating signal peptides from transmembrane regions. *Nat Methods* 8(10):785-786.
2. Moller S, Croning MD, & Apweiler R (2001) Evaluation of methods for the prediction of membrane spanning regions. *Bioinformatics* 17(7):646-653.
3. Janse CJ*, et al.* (2006) High efficiency transfection of< i> Plasmodium berghei</i> facilitates novel selection procedures. *Molecular and biochemical parasitology* 145(1):60-70.
4. Annoura T*, et al.* (2014) Two Plasmodium 6-Cys family-related proteins have distinct and critical roles in liver-stage development. *FASEB J* 28(5):2158-2170.
5. Dearsly AL, Sinden RE, & Self IA (1990) Sexual development in malarial parasites: gametocyte production, fertility and infectivity to the mosquito vector. *Parasitology* 100 Pt 3:359-368.
6. Habtewold T*, et al.* (2019) Streamlined SMFA and mosquito dark-feeding regime significantly improve malaria transmission-blocking assay robustness and sensitivity. *Malar J* 18(1):24.
7. Povelones M*, et al.* (2013) The CLIP-domain serine protease homolog SPCLIP1 regulates complement recruitment to microbial surfaces in the malaria mosquito Anopheles gambiae. *PLoS Pathog* 9(9):e1003623.
8. Winger LA*, et al.* (1988) Ookinete antigens of Plasmodium berghei. Appearance on the zygote surface of an Mr 21 kD determinant identified by transmission-blocking monoclonal antibodies. *Parasite Immunol* 10(2):193-207.
9. Barr PJ*, et al.* (1991) Recombinant Pfs25 protein of Plasmodium falciparum elicits malaria transmission-blocking immunity in experimental animals. *J Exp Med* 174(5):1203-1208.
10. Zavala F, Cochrane AH, Nardin EH, Nussenzweig RS, & Nussenzweig V (1983) Circumsporozoite proteins of malaria parasites contain a single immunodominant region with two or more identical epitopes. *J Exp Med* 157(6):1947-1957.
11. Potocnjak P, Yoshida N, Nussenzweig RS, & Nussenzweig V (1980) Monovalent fragments (Fab) of monoclonal antibodies to a sporozoite surface antigen (Pb44) protect mice against malarial infection. *J Exp Med* 151(6):1504-1513.
12. Dessens JT*, et al.* (1999) CTRP is essential for mosquito infection by malaria ookinetes. *EMBO J* 18(22):6221-6227.
13. Braks JA, Franke-Fayard B, Kroeze H, Janse CJ, & Waters AP (2006) Development and application of a positive-negative selectable marker system for use in reverse genetics in Plasmodium. *Nucleic Acids Res* 34(5):e39.
14. Akinosoglou KA*, et al.* (2015) Characterization of Plasmodium developmental transcriptomes in Anopheles gambiae midgut reveals novel regulators of malaria transmission. *Cell Microbiol* 17(2):254-268.
15. Moon RW*, et al.* (2009) A cyclic GMP signalling module that regulates gliding motility in a malaria parasite. *PLoS Pathog* 5(9):e1000599.
16. Bushell ES*, et al.* (2009) Paternal effect of the nuclear formin-like protein MISFIT on Plasmodium development in the mosquito vector. *PLoS pathogens* 5(8):e1000539.
17. Habtewold T, Povelones M, Blagborough AM, & Christophides GK (2008) Transmission blocking immunity in the malaria non-vector mosquito Anopheles quadriannulatus species A. *PLoS Pathog* 4(5):e1000070.
18. Osta MA, Christophides GK, & Kafatos FC (2004) Effects of mosquito genes on Plasmodium development. *Science* 303(5666):2030-2032.
19. Kim D, Langmead B, & Salzberg SL (2015) HISAT: a fast spliced aligner with low memory requirements. *Nat Methods* 12(4):357-360.
20. Giraldo-Calderon GI*, et al.* (2015) VectorBase: an updated bioinformatics resource for invertebrate vectors and other organisms related with human diseases. *Nucleic Acids Res* 43(Database issue):D707-713.
21. Bahl A*, et al.* (2003) PlasmoDB: the Plasmodium genome resource. A database integrating experimental and computational data. *Nucleic Acids Res* 31(1):212-215.
22. Trapnell C*, et al.* (2010) Transcript assembly and quantification by RNA-Seq reveals unannotated transcripts and isoform switching during cell differentiation. *Nat Biotechnol* 28(5):511-515.
23. Trapnell C*, et al.* (2013) Differential analysis of gene regulation at transcript resolution with RNA-seq. *Nat Biotechnol* 31(1):46-53.
24. Afgan E*, et al.* (2016) The Galaxy platform for accessible, reproducible and collaborative biomedical analyses: 2016 update. *Nucleic Acids Res* 44(W1):W3-W10.
25. Hart T, Komori HK, LaMere S, Podshivalova K, & Salomon DR (2013) Finding the active genes in deep RNA-seq gene expression studies. *BMC Genomics* 14:778.

26. Mi H, Muruganujan A, & Thomas PD (2013) PANTHER in 2013: modeling the evolution of gene function, and other gene attributes, in the context of phylogenetic trees. *Nucleic Acids Res* 41(Database issue):D377-386.
27. Zheng X*, et al.* (2012) A high-performance computing toolset for relatedness and principal component analysis of SNP data. *Bioinformatics* 28(24):3326-3328.
28. Angrisano F*, et al.* (2017) Targeting the Conserved Fusion Loop of HAP2 Inhibits the Transmission of Plasmodium berghei and falciparum. *Cell Rep* 21(10):2868-2878.
